# An engineered baculoviral protein and DNA co-delivery system for CRISPR-based mammalian genome editing

**DOI:** 10.1093/nar/gkae142

**Published:** 2024-02-27

**Authors:** Julien Capin, Alexandra Harrison, Renata A Raele, Sathish K N Yadav, Dominique Baiwir, Gabriel Mazzucchelli, Loic Quinton, Timothy J Satchwell, Ashley M Toye, Christiane Schaffitzel, Imre Berger, Francesco Aulicino

**Affiliations:** School of Biochemistry, University of Bristol, 1 Tankard's Close, Bristol BS8 1TD, UK; School of Biochemistry, University of Bristol, 1 Tankard's Close, Bristol BS8 1TD, UK; School of Biochemistry, University of Bristol, 1 Tankard's Close, Bristol BS8 1TD, UK; School of Biochemistry, University of Bristol, 1 Tankard's Close, Bristol BS8 1TD, UK; GIGA Proteomics Facility, University of Liege, B-4000 Liege, Belgium; Mass Spectrometry Laboratory, MolSys Research Unit, University of Liège, 4000 Liège, Belgium; Mass Spectrometry Laboratory, MolSys Research Unit, University of Liège, 4000 Liège, Belgium; School of Biochemistry, University of Bristol, 1 Tankard's Close, Bristol BS8 1TD, UK; School of Biochemistry, University of Bristol, 1 Tankard's Close, Bristol BS8 1TD, UK; School of Biochemistry, University of Bristol, 1 Tankard's Close, Bristol BS8 1TD, UK; School of Biochemistry, University of Bristol, 1 Tankard's Close, Bristol BS8 1TD, UK; School of Chemistry, University of Bristol, Cantock's Close, Bristol BS8 1TS, UK; Max Planck Bristol Centre for Minimal Biology, Cantock's Close, Bristol BS8 1TS, UK; School of Biochemistry, University of Bristol, 1 Tankard's Close, Bristol BS8 1TD, UK

## Abstract

CRISPR-based DNA editing technologies enable rapid and accessible genome engineering of eukaryotic cells. However, the delivery of genetically encoded CRISPR components remains challenging and sustained Cas9 expression correlates with higher off-target activities, which can be reduced via Cas9-protein delivery. Here we demonstrate that baculovirus, alongside its DNA cargo, can be used to package and deliver proteins to human cells. Using protein-loaded baculovirus (pBV), we demonstrate delivery of Cas9 or base editors proteins, leading to efficient genome and base editing in human cells. By implementing a reversible, chemically inducible heterodimerization system, we show that protein cargoes can selectively and more efficiently be loaded into pBVs (spBVs). Using spBVs we achieved high levels of multiplexed genome editing in a panel of human cell lines. Importantly, spBVs maintain high editing efficiencies in absence of detectable off-targets events. Finally, by exploiting Cas9 protein and template DNA co-delivery, we demonstrate up to 5% site-specific targeted integration of a 1.8 kb heterologous DNA payload using a single spBV in a panel of human cell lines. In summary, we demonstrate that spBVs represent a versatile, efficient and potentially safer alternative for CRISPR applications requiring co-delivery of DNA and protein cargoes.

## Graphical Abstract

**Graphical Abstract:**
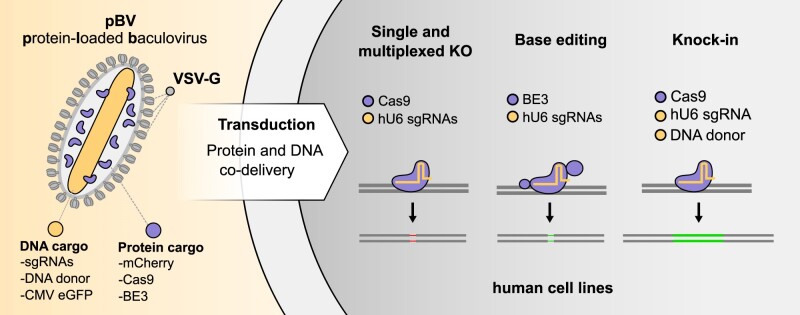

## Introduction

CRISPR technologies have revolutionized gene editing over the past decade, providing highly promising avenues for the treatment of genetic disorders (1–6). Commonly used viral vectors, such as adeno-associated virus (AAV), lentivirus (LV) and adenovirus (AdV) have been largely adopted for the delivery of CRISPR-based genome editing components (7–9). Perhaps with the exception of high-capacity adenoviral vectors (HC-AdV) (9), these are poorly adapted to the new, unique demands of next generation editing technologies, largely due to their limited nucleic acids packaging capacity. Moreover, their propensity for prolonged transgene expression in target cells increases the risk of undesired and often detrimental off-target effects (10–12).

Current efforts to mitigate off-target editing include optimization of sgRNA specificity (13) and the use of high-fidelity SpCas9 variants or orthologs (14–17). Alternative approaches involve delivery of Cas9 protein or pre-assembled Cas9 ribonucleoprotein complexes (RNPs), which reduce the persistence of active enzyme by protein turnover (18). Moreover, protein delivery circumvents the need for transcription and translation activity of genetically encoded elements, resulting in high editing efficacy across different cell types. RNPs have been delivered to cells through a variety of approaches, such as microinjection (19,20) and electroporation (21–23), or by use of nanoparticles (24,25), virus-like particles (26–29) and extracellular vesicles (30–32). However, many of these delivery systems are cytotoxic to cells, expensive to produce, and/or unfit for the co-delivery of heterologous DNA cargo. There is thus an unmet need for alternative systems for simultaneous delivery of protein and DNA cargoes.

We recently demonstrated that baculovirus vectors (BVs), are especially well-suited for the delivery of CRISPR toolkits to human cells (33,34). Indeed, the large circular dsDNA genome (135 kb) of the *Autographa californica* multiple nucleopolyhedrovirus (AcMNPV) is characterized by an unparalleled heterologous DNA cargo capacity. Amongst other applications, this feature enables BV-mediated CRISPR delivery for high-efficiency large DNA knock-in and multiplexed prime-editing in a panel of human cell lines (34). BVs are furthermore replication- and integration-deficient in mammalian cells, two features that make them particularly attractive for therapeutic interventions (35). BVs have been extensively applied to the production of recombinant proteins in insect cells (33,36–38), their natural host, as well as transient delivery of genetically encoded elements to mammalian cells (39–41). Here, we demonstrate that BVs can be effectively repurposed to package and deliver protein payloads, alongside their extensive DNA cargo, to mammalian cells. Using protein-loaded baculovirus (pBV), we achieve successful delivery of active Cas9 protein into mammalian target cells, markedly outperforming gene editing efficiencies obtained by standard plasmid transfection. Furthermore, we demonstrate successful co-delivery of Cas9 protein and heterologous DNA cargo by achieving up to 5% precise genomic insertion of a 1.8 kb heterologous DNA fragment via homology-independent targeted integration (HITI). Importantly, this protein delivery approach is not limited to Cas9, as we further demonstrate that a complete cytidine base editor (BE3) (3) could be loaded into pBVs, resulting in high levels of base editing in target HEK293T cells. Finally, we implement a chemically inducible heterodimerization system to boost selective Cas9 packaging into pBVs (spBVs), achieving superior editing efficiencies in the absence of detectable off-target activity in HEK293T. This approach was ultimately used for efficient multiplexed knock-out and homology-independent targeted integration knock-in in a panel of human cell lines.

## Materials and methods

### Gibson assembly of DNA elements

A full list of all the plasmid sequences used in this study, including assembly information, is provided in Supplementary Table ST1. Plasmid maps and plain .fasta sequences are additionally provided in Supplementary information. Gibson assembly was performed using the NEBuilder HiFi 2× DNA Assembly Mastermix (NEB #E2621S) following manufacturer's recommendations. Briefly, DNA fragments were mixed with backbone to insert ratios of 1:2 (1–2 inserts), 1:1 (> 2 inserts), 1:5 (insert size < 300 bp) to a final volume of 5 μl of ddH_2_O. 5 μl of the 2× master mix was then added to the DNA and incubated at 50°C for 1h. 2 μl of the reaction mix were then used for transformation in homemade electrocompetent Top10 or Pir + *Escherichia coli*. After recovery for 1 h at 37°C in a shaking incubator, the cells were plated on Luria-Bertani (LB)/agar plates with appropriate antibiotics. All precursor plasmids used for Cre-Lox and Multisite gateway assemblies were modified from MultiBac (37,38,42,43) and MultiMate (34) using Gibson assembly. VP39 and P6.9 sequences were PCR-amplified from the isolated MultiBac bacmid and fused to Cas9 by Gibson assembly to create pACE-polh-VP39-Cas9-T2A-mTagBFP-CMV-eGFP, and pACE-polh-P6.9-Cas9-T2A-mTagBFP-CMV-eGFP respectively. To construct the pACE-polH-BE3-CMV-eGFP plasmid, APOBEC-1 and UGI were ordered as synthetic DNA fragments (Eurofins Genomics) and the mutation to generate nCas9(D10A) from Cas9 was introduced by Gibson assembly. pMgK-4–5-polH-VSV-G-ABI, pMgK-5–2-polH-PYL1-Cas9-T2A-GFP and pMgK-1–3-polH-PYL1-mCherryNLS cassettes were assembled using ABI and PYL1 synthetic DNA fragments (Twist Bioscience). The SfU6 promoter was also ordered as a synthetic DNA fragment (Twist Bioscience) and inserted in place of a hU6 promoter on the pACE polH Cas9-T2A-mTagBFP-CMV-eGFP-hU6-sgRNA3 plasmid. The double sgRNA cassette hU6-*HEKs1*-hU6-*HEKs3* was ordered as a synthetic fragment (Twist Bioscience) and cloned into a pMDC donor plasmid. The HDR and HITI-2c payloads were previously described in Aulicino *et al.* 2022 (34) and inserted into pMDK (43) by Gibson assembly.

### Cre-mediated recombination of DNA elements

One acceptor and at least one donor plasmid vector were mixed in equimolar amounts and diluted into a final volume of 10 μl with 0.5 μl of Cre Recombinase (15 000 U/ml, NEB #M0298M), 1 μl of Cre Recombinase Reaction Buffer (provided with Cre recombinase) and ddH_2_O. The reaction was incubated for 1 h at 37°C before heat-inactivation for 10 min at 70°C. The reaction mix was transformed in homemade electrocompetent Top10 *E. coli*, followed by recovery at 37°C for 2 h in a shaking incubator (180 rpm). Cultures were plated on LB agar supplemented with the appropriate antibiotics. *In silico* prediction of Cre-recombination products was done in Cre-ACEMBLER Vers. 2.0 (44,45). A list of Cre-assembled plasmids sequences and precursors is provided in Supplementary Table ST1.

### MultiSite gateway recombination of DNA elements

MultiSite Gateway recombination was carried out as previously described (34). Briefly, one DEST was added to four ENTR vectors in equimolar amounts, each at a final concentration of 2 nM, together with 2 μl of LR Clonase II Plus (Thermo Fisher #12538120), and TE buffer pH 8.0 (Thermo Fisher #12090015) up to 10 μl. Gateway reactions were incubated at 25°C for 16 h. The following day, reactions were stopped by the addition of 1 μl of proteinase K (provided with LR Clonase) followed by a 10 min incubation at 37°C. Reactions were purified and eluted in ddH_2_O before being electroporated into homemade Top10 *E. coli* competent cells. The cells were recovered at 37°C, 180 rpm for 2 h before being plated onto LB agar with suitable antibiotic selection. A list of MSG-assembled plasmids sequences and precursors is provided in Supplementary Table S1.

### Cell culture

Sf21 insect cell cultures (Invitrogen #IPLB-Sf21-AE) were maintained at 0.5–2.0 × 10^6^ cells/ml in ESF 921 culture media (Expression Systems #96-001-01). Cells were kept on a shaker (Thermo Scientific) in 125–250 ml polycarbonate flasks (CORNING, #431143, #431144) at 27°C. Cell counting was performed using a Zeiss Primovert microscope and a haemocytometer (Neubauer).

HEK293T (ATCC #CRL-3216), H4 (ATCC #HTB-148), RPE1-hTERT (ATCC #CRL-4000), SH-SY5Y (ATCC#CRL-2266) and HeLa (ATCC #CRM-CCL-2) were cultured Dulbecco's modified Eagle's media (DMEM) (Gibco #41965), supplemented with 10% foetal bovine serum (FBS) (Gibco #A4766801), 10 U/ml penicillin and 10 μg/ml streptomycin (Gibco #11548876). Primary Huvec (Merck #C-12203) were cultured in Endothelial cell growth medium kit (Merck #C-22110) supplemented with 10 U/ml penicillin and 10 μg/ml streptomycin (Gibco #11548876). A549 (Merck 86012804-1VL) were cultured in Gibco Ham's F-12K (Kaighn's) Medium supplemented (Fisher Scientific #11580556) with 10% FBS (Gibco #A4766801), 10 U/ml Penicillin and 10 μg/ml streptomycin (Gibco #11548876). Cells were kept in 100 mm culture dishes (Gibco) at 37°C in a humified 5% CO_2_ incubator (Thermo Scientific) and passaged by washing with phosphate buffered saline (PBS, Gibco #14190144) and subsequent detachment using 0.25% trypsin (Gibco #25200056). Cells were resuspended in fresh media and split to the desired concentration. Cell counting was performed as previously outlined.

Jurkat (ATCC #TIB-152) were cultured in RPMI-1640 medium (Merck # R8758-500ML) supplemented with 10% heat inactivated FBS (Gibco #A4766801) and 10 U/ml penicillin and 10 μg/ml streptomycin (Gibco #11548876). PBCMs were isolated from blood from waste apheresis material were procured from NHS Blood and Transplant (NHSBT) in accordance with the Declaration of Helsinki and Ethics approval was granted by National Health Service Health Research Authority, Bristol Research Ethics Committee reference 12/SW/0199.

PBMCs were maintained in IMDM (Sigma #FG0465) supplemented with 3 U/ml erythropoietin (Bristol Royal Infirmary), 3 U/ml heparin (Sigma #H3149), 0.2 mg/ml holotransferrin (Sigma # T0665), 3% v/v heat-inactivated Human Male AB Serum (Sigma #H4522), 2 mg/ml Human Serum Albumin (I; Irvine Scientific #9988), 10 μg/ml insulin (Sigma #I9278), 100 U/ml penicillin (Sigma) and 100 μg/ml streptomycin (Sigma), with extra supplementation of 40 ng/ml Stem Cell Factor (SCF; Miltenyi Biotec #130-096-696) and 1 ng/ml IL-3 (R&D Systems #203-IL). Jurkat and PBMCs were maintained in 100 mm culture dishes (Gibco) at 37°C in a humified 5% CO_2_ incubator (Thermo Scientific) and passaged by harvesting and centrifuging at 300 × g for 5 min. After removing spent medium, cell pellets were resuspended in fresh medium, counted as previously described and seeded at the desired density.

### Transfection

HEK293T transfection was performed in a 24–48 multi-well plate using 250–500 ng of plasmid DNA resuspended in TE buffer (Thermo Fisher #12090015) to 5 μl. The DNA was further diluted in 25 μl of a transfection mixture containing PolyFect Transfection Reagent (Qiagen #301105) and Opti-MEM (Gibco #31985062) (1:4) and incubated at room temperature for 15 min before the addition of 100 μl of complete medium. Meanwhile, 2 × 10^5^ HEK293T cells were plated per well in 0.5 ml of complete medium. 125 μl of the DNA-transfection mixture was ultimately added to each well before attachment of the cells. The plate was kept at 37°C in a 5% CO_2_ incubator until phenotype assessment (48–72 h post-transfection).

### pBV and spBV amplification

Assembled plasmids were transformed in chemically competent DH10-MultiBacMam-VSV-G cells comprising an expression cassette for vesicular stomatitis virus glycoprotein used for pseudotyping to enhance transduction (Cas9-pBV, Figure 2) (35), DH10-EMBacY comprising a yellow fluorescent protein (dual Cas9-spBV, Figure 3) (YFP) reporter (37) or commercial DH10Bac (all the other experiments) (ThermoFisher # 10361012) as previously described (38). Plasmids were integrated into the baculovirus genomes by Tn7 transposition following standard protocols (34,37,38,43). After overnight recovery in LB media, cells were plated, and positive colonies selected by blue-white screening. Bacmid extraction was performed using alkaline lysis/ethanol precipitation as previously described (34,37,38,43).

Transfection of the purified bacmid into Sf21 cells, as well as first (V_0_) and second (V_1_) generation viral amplification and harvest were performed as previously described (43). Third generation virus (V_2_) was amplified by infecting 50 ml of cells at 0.8 × 10^6^ cells/ml with 1 ml of V_1_. For abscisic acid (ABA) induced heterodimerization, 50–200 μM of ABA solubilized in 100% ethanol (Generon Bio Basic) were added to the flasks after 24 h. After 3–4 days, cells were pelleted at 4000 x g for 10 min and the V_2_ harvested and passed through a 0.45 μm PVDF filter (Millipore). pBVs were stored at 4°C in the dark until concentration by ultracentrifugation at 80 000 × g for 90 min using SW28Ti rotor (Beckman Coulter). The pellet obtained from 35–38 ml of V_2_ was resuspended in 1 ml of ice cold, sterile PBS (pH 7.4). Concentrated pBV solutions were not filtered and stored at 4°C for up to 2 weeks or supplemented with 5% glycerol for prolonged storage at -80°C. All transductions with pBVs and spBVs described in this manuscript were carried out with viruses processed using the protocol above.

For biochemical characterisation of pBV loading with cargo proteins, density gradient ultracentrifugation was additionally used. Following concentration, pBVs were placed on a 3.7 ml 20–60% discontinuous sucrose gradient (750 μl fractions) and centrifuged at 80 000 × g for 90 min at 4°C using a SW60Ti rotor (Beckman Coulter). Fractions were collected from top to bottom by pipetting and downstream analysis (SDS-PAGE, western blot (15 μl each), DNA extraction (100 μl each) or plate reader fluorescence analysis (100 μl each). For nucleocapsid/envelope fractionation, fractions were collected from the top by pipetting and sucrose removed by overnight dialysis against PBS at 4°C. Nucleocapsid/envelope fractionation was achieved by mixing purified pBVS with 1% NP-40 for 60 min at 4°C, and by subjecting the mixture to the same gradient ultracentrifugation protocol.

### Flow-cytometry

Adherent cells were washed in PBS before detachment with trypsin and resuspension in complete media, supplemented with DRAQ7 (Abcam) (1:1000) to counterstain dead cells. Non-adherent cells were supplemented with DRAQ7 (1:1000). Fluorochromes were detected as follows: eGFP (FITC-A), mCherry (PECF594-A), mTagBFP (BV421-A) and DRAQ7 (APC-Cy7-A). All measurements involving mCherry were performed on a BS LSRFortessa x20 (BD Biosciences) and associated software. Otherwise, either the BS LSRFortessa ×20 or an ACEA Novocyte (Agilent) and associated software were used. Whole cell and single cell populations were identified by forward and side scatters (FSC-A, SSC-A). All FCS files were ultimately analysed on FlowJo (FlowJo LLC) and percentages of fluorescent events were calculated on single, live cells (DRAQ7-). Where shown, percentages of live-cells (DRAQ7-) were gated on single cells.

### pBV and spBV titration by end-point dilution and flow-cytometry analysis

All pBVs and spBVs titrations were performed in HEK293T by end-point dilution and flow-cytometry analysis at 24 h post-transduction as in Aulicino et al. 2022 (34). Briefly, 1 × 10^5^ HEK293T cells were seeded in each well of a 96-wells tissue-culture treated plate (Falcon #353916) in 200 μl/well complete medium. 50 μl of serially diluted viral stocks (typically eight 2-fold dilutions), were added to each well immediately after seeding followed by spinoculation at 600 × g at 27°C for 30 min followed by overnight incubation at 37°C in a humidified incubator with 5% CO_2_. 24 h post-transduction, cells were analysed by flow-cytometry for eGFP expression. eGFP% at each dilution was used to infer a non-linear regression curve in Graphpad Prism. TCID50 and Hill slope were used to calculate TCID10, or the volume of virus sufficient to induce eGFP expression in 10% of the cell culture. Transducing units per ml of viral stock (TU/ml) were calculated by dividing the number of transduced cells (10% of 1 × 10^5^ cells = 1 × 10^4^) by the TCID10 value. For subsequent transduction of target cells at given multiplicity of transductions (MOTs), expected MOTs were calculated as the ratio between TUs and the number of target cells.

### pBV and spBV transduction

1 or 2 × 10^5^ HEK293T cells were seeded per well of a 24 or 48 multi-well plate (Corning), respectively. Before allowing the cells to settle, concentrated pBVs or spBVs were added at the indicated MOTs to each well. To enhance transduction efficiency by spinoculation, the plate was spun at 600 × g for 30 min at 27°C. Thereafter, the plates were kept at 37°C in a 5% CO_2_ incubator. After 24 h, the cells were washed with PBS and the media replaced.

To reduce exposure to pBVs and spBVs, all the other cell lines were transduced with a modified version of the protocol. Briefly, 5 × 10^4^ cells were seeded in 48 multi-well plates (Corning) 24 h prior infection. The next day, pBVs or spBVs were added at the desired MOTs, followed by spinoculation. After spinoculation, the virus was left on the target cells for 4 h. Thereafter, the virus was removed by 2x PBS washes and fresh medium was added. To remove the virus from non-adherent cells, after spinoculation and 4 h incubation with the virus, cells were centrifuged at 600 × g for 5 min. Supernatant was discarded, and cell pellets were resuspended in fresh medium and re-seeded in a new 48-well plate. This transduction protocol applies to Figure 3I-K, Figure 4F-M and relative Supplementary Figure panels.

### Plate reader and Alamar blue assay

For measurement of mCherry content in pBVs following sucrose density gradient fractionation, 100 μl of each fraction were loaded into U-bottom 96-well plates (Falcon #353077).

For measurement of eGFP expression, transduced cells in multi-well plates were analysed in complete media. Raw eGFP relative fluorescence units (RFU) were normalised by subtracting the average signal of untransduced control wells (baseline subtraction).

For the alamarBlue assay, conversion of resazurin (non-fluorescent) to resafurin (fluorescent) in metabolically active cells, was measured at the indicated time-points post-transduction. Before measurement, each well was incubated for 2–4 h with complete media supplemented with 10% alamarBlue reagent (ThermoFisher #DAL1025). Raw RFU were normalised against the average of untransduced control wells and represented as normalised values against their respective controls (100%). eGFP, alamarBlue and mCherry fluorescent spectra were scanned using a a Biotek Synergy H1 (Agilent) using built-in analysis.

### Widefield microscopy

Widefield imaging was performed on a Leica DMI6000 inverted epifluorescence microscope with excitation/emission filters optimized for DAPI, GFP, Rhodamine, Texas Red and Far-Red fluorophores. Images were acquired with a Photometrics Prime 95B sCMOS Camera (1200 × 1200 11 μm pixels) using the Leica LAS-X acquisition software. Cells were maintained at 37°C in the environmental control chamber (Solent).

### Confocal microscopy

Live cells were plated on Lab-Tek borosilicate multi-8 well chamber slides and confocal images were acquired using a Leica SP8 microscope equipped with 405, 458, 476, 488, 496, 514, 561, 594 and 633 nm lasers. Hoechst 33342 (Thermo Fisher #H3570) was added at a final concentration of 1 μg/ml 30 min before imaging to counterstain nuclei.

### Electron microscopy

Copper grids with carbon coating (300 mesh, Electron Microscopy Sciences) were glow discharged for 10 seconds, and 5 μl of purified pBVS resuspended in PBS was placed on the grid for 1 min. The grid was then carefully washed for 10–15 s with PBS and stained by placing it onto a drop of filtered 3% uranyl acetate for 60 s. After each step, the excess solution on the grid was carefully dried using filter paper. Samples were visualized under a FEI Tecnai 20 transmission electron microscope (TEM), and digital micrographs were taken using a FEI Eagle 4K × 4K CCD camera.

### PCR genotyping, Sanger sequencing and deconvolution

All genomic DNA extractions were performed using the PureLink Genomic DNA Mini Kit (Invitrogen), following the manufacturer's instructions. Loci of interest were PCR-amplified using KAPA-2G Fast Genotyping Mix (Sigma), following the manufacturer's recommendations. Briefly, 20–50 ng of gDNA were added to 25 μl of the 2× enzyme mix, 2.5 μl of forward and reverse primers (10 μM), and ddH_2_O up to 50 μl. A 3 min denaturation step at 98°C was followed by 30–40 cycles of denaturation (98°C, 15 s), annealing (60°C, 15 s), and elongation (72°C, 15 s/kb of DNA). A final elongation step at 72°C for 1 min was performed. The PCR product was run on a 1% agarose gel and any bands corresponding to the expected amplicon sizes were gel extracted using the QIAquick Gel Extraction Kit (Qiagen), following the manufacturer's instructions. 15 μl of 5 ng/μl purified PCR product were sent to Eurofins for Sanger sequencing premixed with 2 μl of sequencing primer (10 μM). Sequencing files (.ab1) from control and edited samples were uploaded to Interference of CRISPR Edits (ICE) (Synthego) (46) for trace data deconvolution. sgRNAs and oligonucleotides sequences are provided in Supplementary Tables ST2 and ST3, respectively.

### Quantification of viral genomes by real-time PCR

Baculovirus genomic DNA was extracted from 100 μl (out of 750 μl) sucrose density gradient ultracentrifugation fractions using the NucleoSpin Virus DNA isolation kit (Macherey-Nagel) following manufacturer's recommendations. qPCR reactions were run on a QuantStudio 3 real-time PCR system (Applied Biosystems) in 20 μl/well of a MicroAmp Fast 96-well Reaction Plate (0.1ml) (Applied Biosystems) using 0.5 μl of each primer (10uM), 10 μl of PowerTrack SYBR Green (Applied Biosystems #A46012), 2 μl of the DNA dilutions and 7 μl of ddH20. Forward (CGCTTCACCAACTCTTTGCC) and reverse (AAGAGCTGATCGACCGTTGG) oligos amplifying a portion of gp64 were used as probing oligonucleotides. An activation step of 95°C for 2 min was followed by a 40×x PCR stage of 95°C for 15 s and 60°C for 30 s. Calibration curves were obtained using a serial dilution of a template linearised plasmid containing the gp64 amplicon and all analysis performed using the QuantStudio Design & Analysis software v1.5.2.

### Western blot

SDS-PAGE samples were mixed 3:1 with 4× Laemmli buffer followed by incubation for 5 min at 95°C. Electrophoresis was performed on 3–8% tris-acetate gels (Novex, Thermo Fischer Scientific) using 1× Tris Acetate Buffer (Novex) for 60–80 min at 200V. The PageRuler Plus Protein Standards (Thermo Scientific) were used as the standard molecular mass markers. Gels were transferred to 0.22 μm nitrocellulose or PVDF membranes (BioRad) using the iBlot2 dry blotting system (invitrogen). Membranes were blocked for 1 h at room temperature using 5% skim milk powder in PBS (pH 7.4) with 0.1% Tween20 (PBS-T). The membranes were incubated with primary antibodies (mouse anti-Cas9 (Cell Signalling Technology #14697) (1:1000); mouse anti-Gp64 primary antibody (Santa Cruz Biotechnology #sc-65499) (1:500) and anti-VSV-G Tag (antibodies.com #A121626) (1:1000), overnight at 4°C with gentle agitation. Excess, unbound antibody was removed by washing 3 × 5 min in PBS-T. Membranes were then mixed with anti-mouse HRP-conjugated IgG secondary (Sigma #A5906) (1:2000) or anti-goat HRP conjugated IgG secondary (Santa Cruz Biotechnology #sc-2020) (1:4000) and left at room temperature for 1 h. Excess secondary antibody was washed 3 × 5 min in PBS-T and 3 × 5 min in PBS. The Western was imaged on a gel imaging system (Syngene) after developing the membrane with SuperSignal™ West Pico PLUS chemiluminescent substrate (Thermo Scientific).

### UPLC–ESI-MS/MS

Pellets of concentrated V_2_ were shipped to the GIGA institute (Université de Liège) for proteomic analysis. The samples were dissolved in 200 μl of Tris–HCl pH 8 containing 8 M of urea before sonication for 6 × 10 s in a sonication bath. The protein concentration was determined using the RC DC quantification kit (BioRad) following manufacturer's protocol. Samples were reduced, alkylated, and digested using Lys-C/Trypsin mix (Promega) for 4 h at 37°C. They were then diluted to a 1 M urea concentration and further digested overnight at 37°C. After a quench of the digestion, a 3.5 μg peptide aliquot was purified using a Zip Tip C18 (Merck Millipore). The internal standard MassPrep Digestion Standard Mix (MPDSMix, Waters, Milford, MA, USA) containing 4 digested proteins (BSA P02769, PYGM P00489, ENO P00924, ADH P00330) was spiked in each sample, at a quantity of 150 fmol of ADH digest per injection. 9 μl per sample, corresponding to 1 μg of digested proteins, were analyzed by a LC–MS/MS system composed of an Acquity M-Class UPLC (Waters, Milford, MA, USA) hyphenated to a Q Exactive Plus (Thermo Fisher Scientific, Waltham, MA, USA), used in nanoelectrospray positive ion mode. The protein digests were independently analyzed by LC–ESI-MS/MS. The UPLC trap column was a Symmetry C18 5μm (180 μm × 20 mm) and the analytical column, a HSS T3 C18 1.8 μm (75 μm × 250 mm) (Waters, Milford, MA, USA). The samples were loaded at 20 μl/min on the trap column in 100% solvent A (water 0.1% formic acid) during 3 min and subsequently separated on the analytical column at a flow rate of 600 nl/min, following a linear gradient 0 min, 98% A; 5 min, 93% A; 135 min, 70% A; 150 min, 60% A (solvent A = 0.1% formic acid in water, and solvent B = 0.1% formic acid in acetonitrile), to reach a total run time of 180 min.

The mass spectrometer method applied was a TopN-MSMS method where N was set to 12 (singly charged precursors excluded), meaning that the most intense 12 ions of each MS runs were selected for further MS/MS experiments, then excluded for additional MS/MS selection for 10 seconds. The parameters for MS spectrum acquisition were a mass range from 400 to 1600 *m/z*, resolution of 70 000, AGC target of 1e6 or maximum injection time of 50 ms. The parameters for MS2 spectrum acquisition were isolation window of 2.0 *m/z*, collision energy (NCE) of 28, resolution of 17500, AGC target of 1e5 or maximum injection time of 50 ms.

Databases searches were performed using the SEQUEST HT server and Proteome Discoverer versus 2.1.1.21 (Thermo Fisher Scientific, Waltham, MA, USA), on a chimeric protein database containing: the reviewed Uniprot insect protein sequences (downloaded on the 13-01-2020, 9354 protein sequences); the Autographa californica nuclear polyhedrosis virus database (downloaded on the 13-01-2020, 155 protein sequences); the recombinant protein sequences provided: VSV-G protein (BNNVSVG) and Cas9-T2A-mTagBFP (BNNPI01) and the protein sequences from the commercial standard MPDSMix (Waters).

Carbamidomethyl of cysteines was set as fixed modification, whereas deamidation of asparagine and glutamine and oxidation of methionine were set as variable modifications. Mass tolerances were set at 5ppm for precursors (MS) and 0.02 Da for fragments (MS/MS). The target protein FDR (false discovery rate) was set to 1% and minimum two peptides were required for an identification. Protein relative quantitation was performed by manual integration of the peptides signals by using Skyline software ver. 20.1.0.6. Parameters were selected according to the MS acquisition setup.

### HEK293T stable cell line generation

HEK293T cell lines stably expressing mCherry and sgRNA were obtained by stable transfection of the pL-EF1a -SV40-mCherry-sgRNA-puro plasmids after selection with 1 μg/ml puromycin. Plasmids were transfected as previously described, and after 10 and 20 days of puromycin selection live mCherry positive cells were sorted by Fluorescence-Activated Cell Sorting (FACS) and propagated as polyclonal cell populations. pLKO.1-puro U6 sgRNA BfuAI stuffer (47) was a gift from Rene Maehr & Scot Wolfe (Addgene plasmid # 50920).

## Results

### Baculovirus-mediated delivery of heterologous proteins into human cells

**Figure 1.**
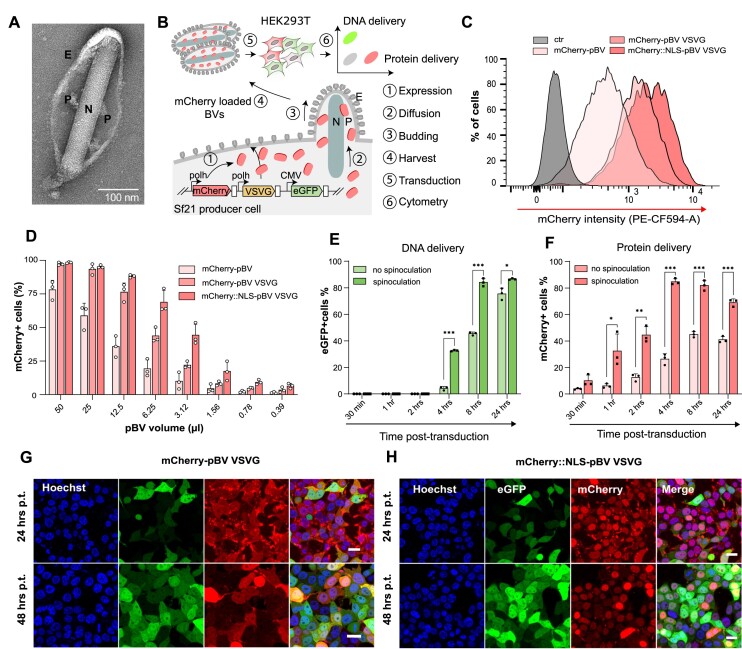
Large pocket formed by the membrane envelope of baculovirus budded virions entraps soluble recombinant proteins overexpressed in insect cells. (**A**) Negative stain imaging of a baculovirus budded virion by electron microscopy (P = Pocket, N = Nucleocapsid, E = Envelope). (**B**) Schematic representation of the experimental setup to validate stochastic protein entrapment in budded virions (BVs) in Spodoptera frugiperda Sf21 cells and subsequent delivery in HEK293T human cells. (C, D) mCherry fluorescence detection in HEK293T 24 h post-transduction with the indicated protein-loaded BV (pBVs) carrying either mCherry or mCherry::NLS protein cargoes. (**C**) Representative flow-cytometry histograms of 10× concentrated pBV and (**D**) percentages of mCherry + cells upon serial-dilutions of concentrated viral stocks. Data are mean + s.d. of *n* = 3 independent biological replicates analysed by flow-cytometry. Multiplicity of infections of undiluted pBV are: mCherry-pBV ≈ 1, mCherry-pBV VSVG ≈ 50 and mCherry::NLS-pBV VSVG ≈ 20. (E, F) Time-course of DNA (eGFP) (**E**) and protein (mCherry) (**F**) delivery in HEK293T transduced with mCherry-pBV VSVG at multiplicity of transduction (MOT) of 50 with or without spinoculation. Data are mean + s.d. of *n* = 3 independent biological replicates analysed by flow-cytometry. (**G, H**) Live confocal microscopy of HEK293T at 24 or 48 h post-transduction with mCherry-pBV VSVG or mCherry::NLS-pBV VSVG at MOT = 10. Hoechst dye counterstains nuclei. Scale bar is 50 μm.

During viral egress, the assembled baculovirus nucleocapsids acquire an envelope consisting of insect cell plasma membrane, producing budded virions (BVs). Electron microscopic analysis of budded virions revealed significant space between the nucleocapsid and the envelope (Figure 1A) with widths reaching up to ∼180 nm, previously defined as an electro-lucent ‘pocket’ (48). We hypothesised that such pockets would likely entrap soluble cytoplasmic proteins during viral budding (Figure 1B). Indeed, early work from Carbonell et al. 1987 (49) demonstrated that virus-borne chloramphenicol acetyl transferase (CAT) activity could be transferred to target cells in the absence of translation and that CAT protein could be detected in viral stocks. Additionally, previous mass spectrometry analysis on BVs revealed the presence of several insect cell and baculoviral-associated host-proteins (50), even though they are not known structural BV proteins or a part of the recently resolved BV nucleocapsid structure (51). Based on these observations, we speculated that BV pockets could potentially be exploited as customizable heterologous protein packaging and delivery tools.

To test this hypothesis, we engineered and amplified a baculovirus carrying a mCherry expression cassette under the control of the strong baculoviral polyhedrin (polH) promoter. An eGFP reporter cassette driven by the CMV promoter was also used to assess transduction efficiency (Figure 1B), enabling the simultaneous quantification of protein (mCherry) or DNA (eGFP) delivery to target mammalian cells. Since pseudotyping the baculovirus with the vesicular stomatitis virus G (VSV-G) protein greatly boosts transduction efficiencies in mammalian cells (40), we also produced and tested VSV-G-pseudotyped BV for protein delivery (pBV VSVG). *Spodoptera frugiperda* Sf21 cells infected by VSV-G pseudotyped baculovirus displayed syncitia formation during the first stage of viral amplification (Supplementary Figure S1A), highlighting increased membrane fluidity and fusogenic potential. Serial dilutions of mCherry-pBV or mCherry-pBV VSVG were then used to transduce HEK293T and the percentage of eGFP and mCherry positive cells were quantified by flow cytometry after 24 h (Figure 1C, D). A third pBV, loaded with mCherry fused to a nuclear localisation signal (mCherry::NLS-pBV VSVG) was used to monitor subcellular trafficking of cargo proteins in target cells.

Next, we sought to investigate if pBVs could be processed and handled similarly to BVs. Indeed, BVs are commonly concentrated and filtered through either .45 or .22 μm filters (52), although their size (in the range of 60 by 300 nm), is ill-suited for commonly used pore size. While marginal viral titre loss was observed after filtration (Supplementary Figure S1B), protein delivery was severely compromised (Supplementary Figure S1C), presumably due to viral envelope shearing and premature protein cargo release. Based on this evidence, filtration was not included for subsequent experiments.

To exclude that the mCherry signal in target cells was the result of polH promoter activity, plasmids carrying CMV eGFP or polH mCherry were transfected in HEK293T. 48 h after transfection, contrary to the CMV eGFP plasmid, no fluorescence was detected in cells transfected with the polH mCherry construct (Supplementary Figure S1D). To further exclude other protein-delivery mechanisms unrelated to BV, we used sucrose density gradient purification to isolate pBVs from other soluble proteins or contaminants (Supplementary Figure S1E). Fractions containing pBVs were readily identifiable on SDS-PAGE through the presence of gp64, VSVG and vp39 (Supplementary Figure S1F). mCherry fluorescence was exclusively detected in fractions enriched for viral particles (Supplementary Figure S1G), demonstrating that the protein cargoes are strongly associated with pBVs.

All the tested pBVs displayed high DNA delivery (eGFP%) and protein delivery (mCherry%) in target cells, with a marked improvement when pBVs were pseudotyped with VSV-G (Figure 1C,D, Supplementary Figure S1H,I). At the same time, no decrease in cell viability was observed in HEK293T at 24 h post-transduction, irrespective of the viral volume used (Supplementary Figure S1J).

Compared to the delivery of genetically encoded elements, protein delivery is typically faster, since no nuclear incorporation, transcription or translation processes are required. To have a side-by-side comparison of DNA and protein delivery kinetics, HEK293T were exposed to mCherry-pBV VSVG for up to 24 h, with or without initial spinoculation. While eGFP (DNA delivery) started to be expressed 4 h post-transduction, with a peak at 24 h (Figure 1E), mCherry (protein delivery) was immediately detected 30 min post-transduction, with a peak at 4–8 h and a reduction at 24 h (Figure F). Both processes were markedly improved by spinoculation, with higher eGFP and mCherry percentages (Figure 1E, F) and higher intensities of mCherry fluorescence (Supplementary Figure S1K).

Finally, we sought to assess whether the delivered protein cargoes could be targeted to specific subcellular compartments such as the nucleus. HEK293T were transduced with mCherry-pBV VSVG or mCherry::NLS-pBV VSVG and imaged at 24 or 48 h post-transduction. While mCherry was ubiquitously distributed within cells (Figure 1G), mCherry::NLS was markedly enriched in the nuclear compartments (Figure 1H). Alongside the correct subcellular localisation pattern, both pBVs displayed punctate intracellular mCherry patterns at 24 h post-transduction (Figure 1G, H, top panels) which were almost entirely absent at 48 h post-transduction (Figure 1G, H, bottom panels). This could potentially reflect the endosomal trafficking of pBV-delivered protein cargoes after internalisation.

To standardise pBV stocks thereafter, we adopted titration by end-point dilution and fluorescence measurement in HEK293T (Supplementary Figure S1L). In this manner, we could obtain functional virus titres (or transducing units, TU) to characterise DNA delivery, protein delivery and viability against known multiplicities of transduction (MOTs) (Supplementary Figure S1M). Finally, to test whether protein cargo delivery via pBV was restricted to HEK293T, we transduced SH-SY5Y cells, a human neuroblastoma cell line, with mCherry::NLS-pBV VSVG. 48 h after transduction, mCherry signal was predominantly localised with the nuclear comparments, alongside residual cytoplasmic mCherry signal (Supplementary Figure S1N).

Taken together, these results demonstrate that pBVs can effectively deliver protein cargoes to target mammalian cells with fast internalisation dynamics. Additionally, pBV applications are not restricted to easy-to-transfect cell lines and the delivered protein cargoes retain their normal subcellular localisation properties. This makes them an attractive tool for the delivery of RNA-programmable CRISPR-associated proteins.

### pBV-mediated delivery of Cas9 enables rapid and efficient editing in mammalian cells

**Figure 2.**
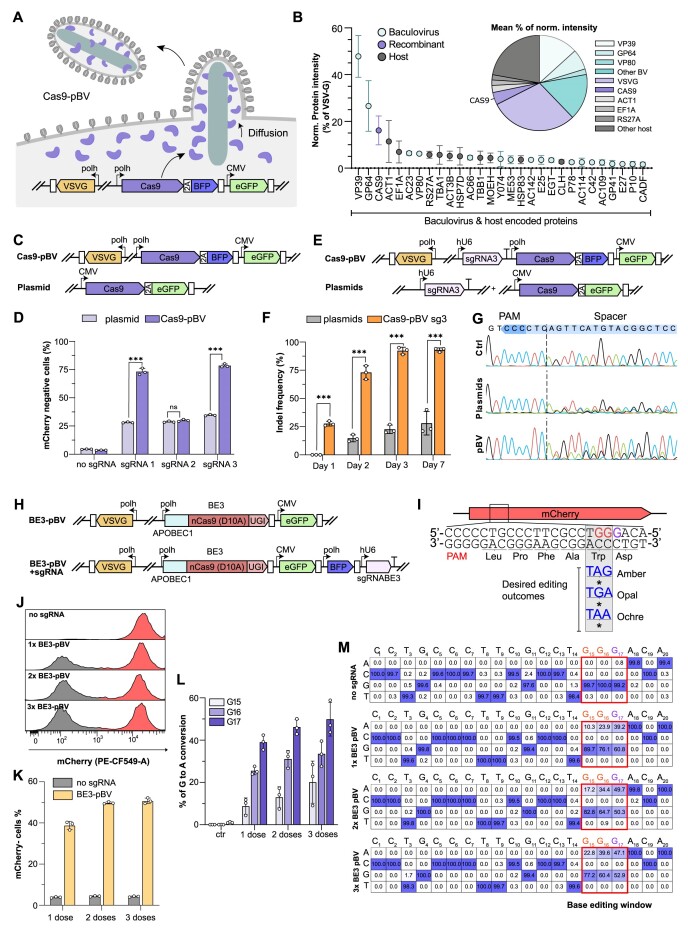
Overexpressed Cas9 is abundant in pBV and enables rapid and highly efficient genome editing in HEK293T. (**A**) Schematic representation Cas9-pBV production in Sf21 insect cells. (**B**) Histogram of protein intensities normalised to VSVG (top 30 most abundant proteins identified in concentrated Cas9-pBV by UPLC–MS/MS) and pie-chart of discovered proteins abundancies. Colours indicate recombinant (purple), baculovirus (turquoise) and host (grey) encoded proteins. Mean ± s.d. of *n* = 3 independent biological replicates. (C, D) Comparison of mCherry knock-out efficiencies in HEK293T stably expressing mCherry and mCherry targeting sgRNAs following transfection or transduction with the indicated (**C**) Cas9 encoding plasmid or Cas9-pBV. (**D**) mCherry- cells % at 7 days post-transfection (500 ng plasmid) or Cas9-pBV transduction (MOT = 50). Data are mean + s.d. of *n* = 3 independent biological replicates analysed by flow-cytometry. *** *P* < 0.001, ns = not significant, Student's *t*-test. (E-G) Indel formation dynamics in HEK293T stably expressing mCherry following transduction or transfection with plasmids or Cas9-pBV indicated in (**E**). (**F**) Indels formation at the indicated time points following transfection or transduction with plasmids or Cas9-pBV and (**G**) representative Sanger sequencing chromatograms at 7 days. Data are mean ± s.d. of Sanger sequencing decomposition analysis (ICE) of *n* = 3 independent biological replicates. *** *P* < 0.001, Student's *t*-test. (H–M) mCherry knock-out via base editors delivered through pBV in HEK293T stably expressing mCherry. (**H**) Schematic representation of BE3 pBV with or without sgRNA and (**I**) overview of the targeted mCherry region for the introduction of a stop codon using BE3. In the editing window target and non-target G-nucleotides are highlighted in red and purple, respectively. (J-K) Flow-cytometry analysis of mCherry loss at 7 days post-transduction with 1, 2 or 3 consecutive administrations of PE3-pBV + sgRNA each at MOT ≈ 50. (**J**) Representative flow-cytometry histograms and (**K**) percentages of mCherry- cells. Mean ± s.d. of *n* = 3 independent biological replicates. (L-M) G-to-A conversion efficiencies in the editing window (**L**) and representative individual base editing rates (**M**). Data are mean ± s.d. of Sanger sequencing data analysed with EditR. *n* = 3 independent biological replicates.

To confirm if Cas9, similarly to mCherry, could be loaded into pBVs and delivered to target cells, we engineered Cas9-pBVs including a Cas9-T2A-mTagBFP under the control of a polH promoter. As previously, a CMV driven eGFP cassette was included as a DNA cargo delivery detection module (Figure 2A). Compared to mCherry (28 kDa), Cas9 has a much larger molecular weight (163 kDa) which could potentially be detrimental for pBV loading. To confirm pBV loading with recombinant Cas9, concentrated Cas9-pBVs were fractionated via sucrose density gradient ultracentrifugation (Supplementary Figure S2A). Cas9 protein was strongly associated with fractions enriched for pBVs, as confirmed by quantification of viral genomes and western blot analysis of Cas9, gp64 and VSV-G across sucrose density fractions (Supplementary Figure S2A-C). For a representative Cas9-pBV stock (≈3 × 10^8^ TU/ml), the Cas9 protein content was ≈400 ng/ml, as quantified by a western blot calibrated with known Cas9 purified protein standard (Supplementary Figure S2D, E).

Next, Cas9-pBVs were purified by concentration and their content analysed by UPLC-MS/MS to detect the relative abundance of the proteins of interest in the viral particles (Figure 2B, Supplementary Table ST2). A total of 232 unique proteins were identified in at least two of the three replicates, corresponding to 69 baculovirus-encoded proteins and 163 insect cell proteins (Figure 2B, Supplementary Figure S2F). Out of these, the envelope VSV-G protein was the most abundant, followed by VP39, the major nucleocapsid protein and GP64, the main baculovirus envelope glycoprotein (53). Relative quantitation of protein content based on the Hi-Top3 method (54) detected Cas9 as the fourth most abundant protein in Cas9-pBVs, representing ∼16% of the VSV-G intensity (Figure 2B, Supplementary Figure S2F). As anticipated, insect cell host proteins were also found in pBV samples, collectively accounting for ∼28% of the relative quantity of proteins detected. These included naturally abundant insect proteins associated with nucleocapsid egress, such as actin and tubulin isoforms, as well as membrane-localized proteins such as clathrin and moesin/erzin/radixin homolog 1 (MEOH). Interestingly, other soluble proteins were found in significant amounts such as the translation elongator factor 1 alpha (EF1A), the ribosomal protein S27A (RPS27A) and heat shock proteins 7D and 83 (HSP7D; HSP83). These proteins are all highly expressed in insect cells and are not known to interact with components of the BV, which further supports our hypothesis of stochastic protein entrapment during viral amplification and budding.

We next sought to assess the potential of Cas9-pBVs for genome engineering applications. For this, we developed a simple mCherry knock-out (KO) assay to monitor gene editing efficiencies via a fluorescence-based readout. First, we generated four HEK293T cell lines stably expressing mCherry with or without three different mCherry targeting sgRNAs. These were transfected or transduced with Cas9 expressing plasmids or Cas9-pBVs, respectively (Figure 2C). DNA delivery efficiencies, mCherry fluorescence and cell viability (Supplementary Figure S2G–I) were monitored at early time points after transfection/transduction. mCherry levels monitored at 24/48 h post-transfection were unchanged (Supplementary Figure S2H), presumably due to its high protein stability. KO events were detectable via flow-cytometry after 10 days for both plasmids and Cas9-pBVs. While plasmid transfection resulted in up to 34% mCherry KO, baculovirus-delivered Cas9 protein achieved up to 77% KO efficiency (Figure 2D, Supplementary Figure S2J), markedly outcompeting plasmid transfection and confirming that Cas9 protein could not only be efficiently packaged, but also retained its activity when delivered using pBVs.

So far, pBVs were only engineered to deliver protein cargoes, while their DNA delivery capacity was exploited to monitor transduction rates and dynamics. Indeed, DNA constructs could be engineered to express *cis-*acting sgRNAs, to generate all-in-one pBVs able to delivery Cas9 protein and express sgRNAs in target cells.

For this reason, we engineered a Cas9-pBV encoding an mCherry-targeting sgRNA driven by the human U6 promoter (hU6-sgRNA) (Figure 2E) and investigated gene editing efficiencies and dynamics in mCherry HEK293T stable cells. Gene editing dynamics were monitored every 24 h for 3 days and after 7 days through Sanger sequencing deconvolution (Inference of CRISPR Edits, ICE) (46). Co-transfection of sgRNA and Cas9 expressing plasmids (Figure 2E) was performed side-by-side for comparison. All-in-one Cas9-pBV showed overall faster dynamics and higher indel generation efficiencies compared to plasmid co-transfection (Figure 2F,G). Indeed, indels were detected as early as 24 h post-transduction with Cas9-pBVs, while 48 h were required to produce the first detectable indels in transfected samples (Figure 2F). This is likely due to the transcription/translation latency of the genetically encoded Cas9 and sgRNA.

Although protein delivery should act even faster (Figure 1F), we could not detect indels before 24 h in mCherry HEK293T transduced with Cas9-pBVs (data not shown). Once again, this is possibly due to the transcription latency of the genetically encoded hU6 sgRNA.

In terms of absolute editing efficiency, Cas9-pBVs outperformed plasmid transfection, giving a ∼3.8-fold higher maximum indel frequency up to 95% at 7 days post-delivery (Figure 2F,G and Supplementary Figure S2J).

Thus, recombinant Cas9 protein and genetically encoded sgRNAs could be successfully co-delivered for targeted DSB induction, displaying both higher gene editing activity and faster dynamics when compared to plasmid transfection.

Building on these results, we sought to assess whether pre-assembled Cas9/sgRNA RNPs could be loaded into pBVs instead of relying on a genetically encoded module for sgRNA expression in target cells. To produce RNP-pBVs, we used a previously characterized *Spodoptera frugiperda* U6 promoter (SfU6) to transcribe the sgRNA in insect cells (Supplementary Figure S2K). While RNP-pBVs could elicit up to around 20% of mCherry KO in target cells, their efficiency was greatly reduced compared to all-in-one Cas9-pBVs with a genetically encoded hU6-sgRNA cassette (Supplementary Figure S2L). It is possible that the nuclear localisation of U6-transcribed sgRNAs restricts their loading into pBVs during the budding process, as was previously reported in mammalian cells during the development of other RNP delivery systems (29,31). Interestingly, most of the editing efficiency could be recovered when RNP-pBVs were used to transduce cells stably expressing the mCherry-targeting sgRNA3, indicating that the sgRNA packaging is the main limiting factor in RNP-pBVs (Supplementary Figure S2L). Despite the modest editing efficiency, these results lay the foundation for the future RNP-pBVs generation, while providing the first evidence that a single BV can simultaneously deliver heterologous protein, RNA and DNA to human cells.

### pBVs support cytidine base editor protein delivery for efficient base conversion

More recent CRISPR-based approaches such as base editing (BE) (3,4) and prime editing (PE) (5,6) afford precision engineering without the requirement for double-strand breaks (DSBs), alleviating DSB-related safety concerns. Nonetheless, BE can induce Cas-dependent and Cas-independent off-target effects, with the latter caused by overexpression of the cytidine deaminase (55–57). Similar to Cas9-dependent off-targeting, it has been demonstrated that unspecific base conversion caused by the cytidine deaminase can be alleviated using protein delivery methods (26). We therefore investigated whether our baculovirus-mediated protein delivery approach could be harnessed for the delivery of an entire cytidine base editor (BE3) (Figure 2H). A validated sgRNA targeting a tryptophan (W63) residue (TGG) of the mCherry gene was used to produce a C-to-A conversion on the non-coding strand. In this manner, the TGG codon could be converted to TAG (Amber), TGA (Opal) and TAA (Ochre) stop codons (58) (Figure 2I). BE3-pBVs with or without the sgRNA cassette were amplified and used to transduce HEK293T cells stably expressing mCherry. Up to three BE3-pBVs doses were sequentially applied to transduce reporter cells, with at least five days between each dose (Figure 2J-M). Unlike DSB-based approaches, for which the most likely outcome is indel formation resulting in the disruption of the protospacer, base editing efficiency can be enriched by successive treatment, as the protospacer sequence is rarely altered in the absence of correct editing. Promisingly, a single dose of BE3-loaded vector was already sufficient to yield up to ∼40% of mCherry negative cells, while one or two additional doses further increased mCherry loss up to ∼50% (Figure 2J,K). To verify whether mCherry disruption was a consequence of the intended base editing outcomes at the DNA level, Sanger sequencing deconvolution analysis with EditR (59) was performed on all samples (Figure 2L,M). As expected, the G15 and G16 nucleotides from the TGG codon were effectively converted to A in a significant proportion of all sequences. Unsurprisingly, the G17 nucleotide present in the BE3 editing window was also largely converted to adenine, leading to the missense mutation D64N. If this mutation leads to a functional mCherry while altering the protospacer sequence, it may partly explain the modest enrichment in edited cells observed after successive dosing.

These results confirm that functional proteins of a wide range of sizes (mCherry 28kDa, Cas9 160kDa and BE3 198kDa) can be effectively packaged into pBV for delivery. We anticipate that efficient delivery of even larger protein machineries such as prime editors (5,6), CAST (60), or combinations thereof, might be feasible using pBV.

### Improving selective protein loading into pBVs enhances genome editing efficiency without detectable off-target effects

**Figure 3.**
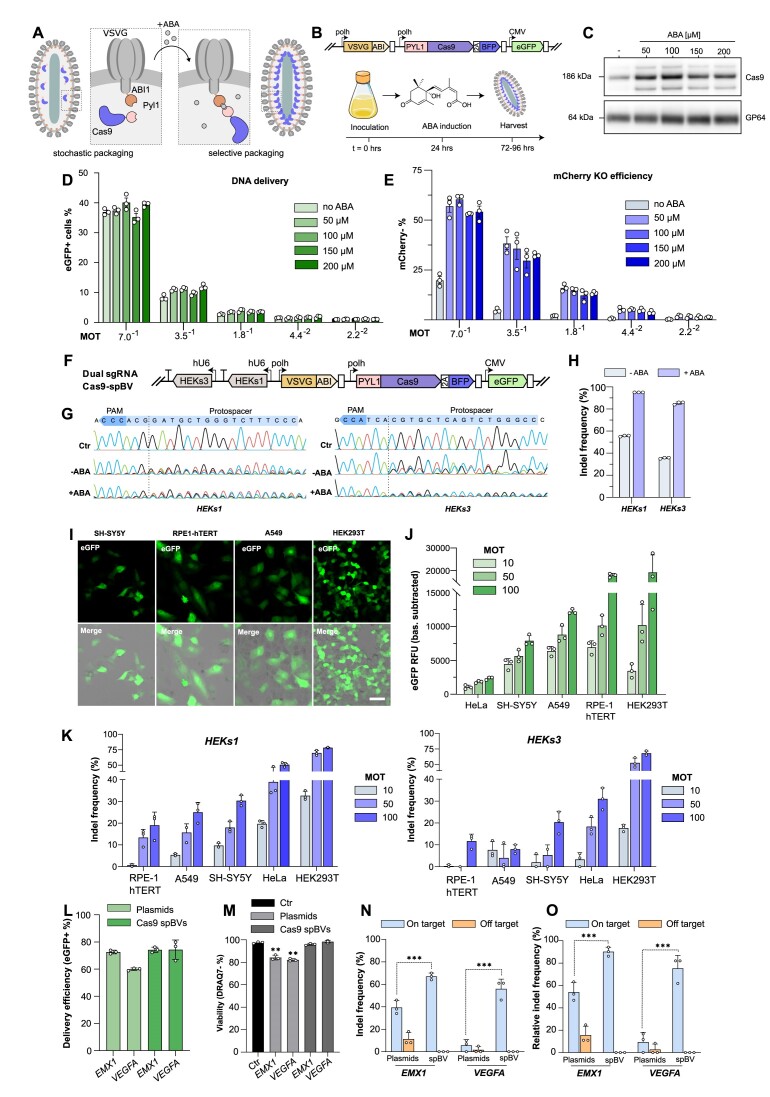
Selective incorporation of Cas9 in pBV (spBV) leads to higher editing efficiencies in a panel of human cell lines while maintaining undetectable off-target editing. (A-C) Development of an abscisic acid (ABA) inducible selective protein packaging strategy. (**A**) Pyl1 and ABI1 domains are fused to the N-terminal and C-terminal domain of Cas9 and VSVG, respectively. During viral packaging, addition of ABA promotes selective packaging of Cas9 into budded virions. (**B**) Workflow of ABA-induced Cas9-spBV production in Sf21 insect cells. (**C**) Western blot of Cas9 and gp64 in concentrated spBV cultured in presence or absence of ABA at the indicated concentrations [μM]. (D, E) DNA delivery and mCherry KO efficiencies in HEK293T stably expressing mCherry and sgRNA 3 following transduction with serial dilution of Cas9 spBV amplified in presence or absence of ABA at the indicated concentrations. (**D**) % of eGFP + cells assessed 24 h post-transduction and (**E**) mCherry KO efficiency assessed at 10 days post-transduction. MOT = multiplicity of transduction. Histogram of flow-cytometry data. mean ± s.d. of *n* = 3 independent biological replicates. (**F**) Schematic representation of multiplexed genome editing approach via spBV in HEK293T using a single Cas9-spBV carrying two sgRNA cassettes (*HEKs1* and *HEKs3*). (G-H) Indel frequencies at *HEKs1* and *HEKs3* loci in HEK293T transduced with dual sgRNA Cas9-spBV at MOT 200 amplified in presence or absence of 100 μM ABA. (**G**) Representative Sanger sequencing chromatograms and (**H**) histogram of Sanger sequencing deconvolution data (ICE), n = 3 independent biological replicates. (I–K) Multiplexed spBV editing in HeLa, SH-SY5Y, RPE1-hTERT, A549 and HEK29T cells. (**I**) Live confocal microscopy at 24 h post-transduction (scalebar is 50 μm); (**J**) eGFP expression levels at 24 h post-transduction, mean ± s.d. of *n* = 3 independent biological replicates. (**K**) Indel frequencies at *HEKs1* and *HEKs3* loci in the indicated cell lines transduced with dual sgRNA Cas9-spBV amplified in presence of 100 μM ABA at MOT 10, 50 and 100. Data are mean + s.d. of Sanger sequencing deconvolution data (ICE), *n* = 3 independent biological replicates. (L–O) On-target and off-target indels frequencies in HEK293T following co-transfection with CMV Cas9 + individual sgRNA plasmids (500 ng) or all-in-one Cas9-spBV equipped with either *EMX1* or *VEGFA* sgRNAs (MOT 10). (**L**) DNA delivery efficiency and (**M**) DRAQ7- cells (viability) at 48 h post-transfection or 24 h post-transduction. Histograms of flow-cytometry data, mean ± s.d. of n = 3 independent biological replicates. ** *P* < 0.01, Student's *t*-test. (N, O) Indel frequencies at on- and off- targets sites at 72 h post-transfection or transduction. (**N**) Absolute indel frequencies and (**O**) Normalised indel frequencies relative to transduction efficiencies in (L). Histograms of Sanger sequencing deconvolution data (ICE), mean + s.d. of *n* = 3 independent biological replicates. *** *P* < 0.001, Student's *t*-test.

So far, proteins of interest were loaded into pBVs by stochastic entrapment. Although this approach enabled high levels of genome editing, we reasoned that engineering an active and selective protein loading system could further increase the potency of pBVs. We first attempted to fuse Cas9 to abundant baculovirus capsid proteins such as VP39 or P6.9 to predominantly localize Cas9 to- or within the nucleocapsid, respectively (Supplementary Figure S3A). We confirmed by western blot that the protein fusions were incorporated into the pBVs (Supplementary Figure S3B), with the VP39-Cas9 fusion more effectively incorporating Cas9 in the capsids than P6.9-Cas9 (Supplementary Figure S3C). Despite successful incorporation of the proteins into pBVs, upon protein delivery, VP39- and P6.9-Cas9-pBVs showed significantly reduced editing efficiencies compared to standard Cas9-pBVs (Supplementary Figure S3D-F). This may be a consequence of VP39- and P6.9-Cas9 fusion proteins being tethered to the viral capsids in target cells, hindering their trafficking to the nucleus. We then reasoned that maximising the local concentration of Cas9 near the cell envelope could result in increased entrapment into pBVs. At the same time, we anticipated that fusing Cas9 to membrane proteins without a release mechanism would likely abolish their nuclear import in target cells. To enable membrane tethering in insect cells and release in target cells, we opted for the chemically inducible ABI/PYL1 dimerization system, which is reversible upon withdrawal of the inducer abscisic acid (ABA) (61). We created a loading module by fusing ABI to the cytoplasmic C-terminal tail of VSV-G, while the protein cargoes were fused to PYL1 at their N-terminus (Figure 3A, Supplementary Figure S3G). We hereafter refer to pBVs generated using this inducible, active packaging system as spBVs (selective protein loaded baculoviruses).

We first tested spBVs using mCherry::NLS as the selective protein cargo (Supplementary Figure S3G-K). ABI/PYL1 heterodimerization was induced by adding 100 μM of ABA 24 h after infection of insect cell cultures. spBVs amplified in the presence of ABA, effectively improved mCherry delivery to target HEK293T cells between 4.3- and 5.3-fold as compared to standard pBVs (Supplementary Figure S3H,I). Increased levels of mCherry delivery were also observed without ABA treatment, potentially suggesting a basal level of heterodimerization in the absence of the inducer (Supplementary Figure S3I). As a consequence of increased protein loading per virion, spBVs effectively induced higher mCherry incorporation rates at much lower viral doses (Supplementary Figure S3J, K).

Encouraged by these results, we tested whether spBVs could also improve gene editing efficiencies. 50, 100, 150 and 200 μM ABA were added to insect cell cultures during Cas9-spBV production (Figure 3B). Western blot analysis of concentrated spBV stocks confirmed selective Cas9 enrichment at all tested ABA concentrations (Figure 3C).

Next, we tested the impact of Cas9-spBVs on gene editing efficiencies. HEK293T stably expressing mCherry and the mCherry targeting sgRNA3 were transduced with serial dilutions of titre-normalised Cas9-spBV amplified in the presence of a range of ABA concentrations (Figure 3D, E). While all the spBVs resulted in comparable DNA delivery efficiencies (Figure 3D), Cas9-spBVs amplified in the presence of any ABA concentration led to substantially higher levels of genome editing as compared to the non-induced control (Figure 3E). Indeed, across the range of dilutions tested, ABA-induced Cas9-spBVs exhibited from ∼3- to 8-fold higher editing efficiencies, confirming that the increased protein loading translates into higher protein delivery and gene editing activity in target cells.

We then challenged our chemically induced enrichment approach to perform multiplexed gene editing using a single Cas9-spBV. We designed a dual sgRNA Cas9-spBVs, targeting the previously validated *HEKs1* and *HEKs3* loci (62) in HEK293T (Figure 3F). Indel generation 72 h post-transduction at both loci was taken as a measure of editing activity. At both loci, simultaneously edited using a single spBV, higher gene editing rates were observed when viruses were amplified in the presence of 100 μM ABA, with indels frequency increasing from 55% to 95% (*HEKs1*) and 35% to 85% (*HEKs3*) (Figure 3G,H).

To fully validate spBV-mediated Cas9 protein delivery, we finally transduced a panel of human cell lines with the dual Cas9-spBV targeting *HEKs1* and *HEKs3*. As previously, the spBVs were amplified in the presence of 100 μM ABA. In addition to HEK293T, HeLa (cervical carcinoma), SH-SY5Y (human neuroblastoma), A549 (human lung carcinoma), and RPE1-hTERT (human retinal pigmented epithelial cells, hTERT immortalised) were transduced with increasing doses of the all-in-one Cas9-spBV. Informed by pBV transduction and protein delivery dynamics (Figure 1E, F), we modified the transduction protocol to minimise the exposure to VSV-G, whose toxicity has been well documented (63). This involved removing the spBVs from the target cells 4 h after spinoculation.

Transduction was monitored via microscopy and fluorescence readout of eGFP expression (Figure 3I,J), while editing efficiencies were measured using Sanger sequencing deconvolution of the target cells harvested at 72 h post-transduction.

Dose-dependent indels were detected at both loci in all cell types, albeit with varying degrees of efficiency (Figure 3K), demonstrating that baculovirus-mediated protein delivery is a viable technology for protein and DNA delivery in human cells. Interestingly, gene editing efficiency was not directly linked to spBV ability to induce eGFP expression. For instance, poorly transduced cells (e.g. HeLa) displayed very high gene editing efficiencies, while cell lines with better transduction rates (e.g. RPE1-hTERT) displayed lower indel frequencies (Figure 3J, K). This finding potentially highlights cell-type dependent mechanisms of protein and DNA cargoes processing following cell-entry, such as innate immune responses or protein trafficking. Additionally, spBVs were overall well tolerated by recipient cells, with moderate MOT-dependent decrease in viability at 48 h observed only in A549 and RPE1-hTERT (Supplementary Figure S13L).

Given the high on-target editing efficiencies induced by Cas9-spBVs, we next sought to evaluate their off-target editing effects. One of the advantages of Cas9 protein delivery is a significant reduction in off-target site cleavage (28,30), generally representing a safer approach than genetically encoded Cas9 delivery. To evaluate off-target activity, we engineered two individual Cas9-spBVs to target the *EMX1* and *VEGFA* loci using previously validated sgRNAs with characterized off-target editing rates (30). Indel formation at the *EMX1*, *VEGFA* and at their respective off-targets loci was assessed 72 h post transduction using Sanger sequencing deconvolution. These results were compared to indel generation achieved by plasmid transfection using the same sgRNAs. HEK293T were once again chosen as the target cells, having previously displayed the highest transfection, transduction and editing efficiencies. The amount of spBVs used was adjusted to obtain DNA delivery efficiencies comparable to those obtained by plasmid transfection (Figure 3L). Nonetheless, a significant decrease in viability at 48 h was observed for plasmid transfection but not for spBV transduced cells (Figure 3M). As previously, the absolute and relative on-target indel rates were higher for spBV transduced cells while, in marked contrast to plasmid transfection, off-target indels were undetectable. (Figure 3N,O).

Taken together, our results establish that Cas9 protein loading into pBVs can be selectively increased by using synthetic, drug-inducible heterodimerization domains. The increased protein loading and delivery directly resulted in higher editing efficiencies, enabling single and multiplexed gene editing approaches in a panel of human cell lines. Importantly, spBVs were well tolerated, with little reduction in viability in a subset of target cells. Finally, notwithstanding higher Cas9 loading and high on-target efficacies, corresponding off-target activities were undetectable using our approach, highlighting the superior safety of spBV-mediated Cas9 protein delivery and its applicability across different human cells.

### Cas9-pBVs support large heterologous DNA cargo delivery for targeted integration

**Figure 4.**
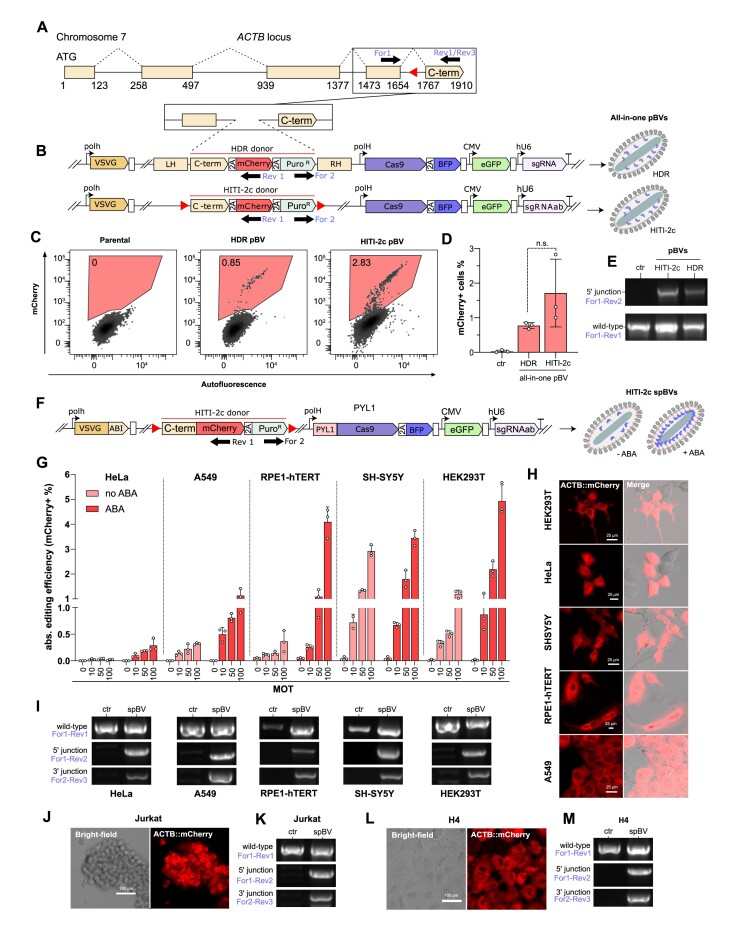
Knock-in using all-in-one pBV or spBV in diverse cell types. (A–E) pBV mediated *ACTB* C-terminal exon tagging via HDR or HITI-2c strategies. (**A**) Schematic representation of human *ACTB* locus. Red-triangles represent position and orientation of the selected *ACTB* sgRNA target site. (**B**) Constructs used to generate all-in-one HDR or HITI-2c pBV differ only for the DNA donor. The HDR donor is equipped with homology arms, while the HITI-2c donor is flanked by sgRNA targeted sequences. The knock-in cassette encodes a synthetic C-terminal exon fused to T2A::mCherry::P2A::Puro-R. Genotyping oligonucleotides are indicated by black arrows in (A, B and F). (C-D) mCherry detection in HEK293T 5 days post-transduction with the indicated all-in-one HDR or HITI-2c pBV at MOT 100. (**C**) Representative flow-cytometry dot plots and (**D**) histogram of flow-cytometry data. Mean ± s.d. of n = 3 independent biological replicates. n.s.=not significant, Student's *t*-test. (**E**) PCR genotypes of puromycin selected HEK239T transduced with all-in-one HDR or HITI-2c with 5′ junction or wild-type allele specific primer pairs. (F-M) spBV mediated *ACTB* C-terminal exon tagging via HITI-2c in a panel of human cell lines. (**F**) Construct used to generate all-in-one HITI-2c pBV. The knock-in cassette encodes a synthetic C-terminal exon fused to mCherry::T2A::Puro-R. (**G**) Knock-in efficiencies in HeLa, A549, RPE-1 hTERT, SH-SY5Y and HEK293T at 48 h post-transduction with HITI-2c-spBV at the indicated MOTs, amplified in presence or absence of 100 μM ABA. Data are mean + s.d. of flow-cytometry data. *n* = 3 independent biological replicates. (**H**) Representative live confocal images of unselected HITI-2c spBV ABA transduced cells at MOT 100 at 48 h post-transduction. Scalebar is 25 μm. (**I**) PCR genotype with wild-type, 5′ junction and 3′ junction specific primer pairs following puromycin selection for 4 days and amplification in absence of selective pressure for 15 days (HeLa, A549, SH-SY5Y and HEK293T), or at 4 days post-transduction in absence of selective pressure (RPE-1 hTERT). **(J–M)** Live confocal imaging and PCR genotype of puromycin selected Jurkat (J-K) and H4 (L-M) cell lines following transduction with HITI-2c spBV + ABA at MOT 10 and 100, respectively. Scalebar is 100 μm.

After demonstrating high levels of gene disruption using Cas9-pBVs, we next challenged our delivery vector to produce a targeted insertion of co-delivered heterologous DNA cargo by harnessing homology-directed repair (HDR) or non-homologous end-joining (NHEJ) DNA repair pathways. We previously demonstrated efficient large DNA cargo docking in human genomes using a single baculovirus, which relied on genetically encoded Cas9, guide RNAs and DNA donor (34). Using the same approach, we designed a 1.8 kbp DNA insert, containing the last C-terminal exon of the β-actin (*ACTB*) gene fused to mCherry followed by a puromycin selection marker, spliced by T2A and P2A self-cleaving peptides, respectively (Figure 4A, B). Two all-in-one Cas9-spBVs vectors were designed to achieve targeted DNA integration via homology-directed repair (HDR) or homology-independent targeted integration (HITI-2c) (Figure 4B). The HDR donor was flanked by ∼1kb left and right homology arms, spanning the genomic cleavage site, while the HITI-2c donor was flanked by two identical sgRNA target sites in reverse orientation to their genomic counterpart (64). Cleavage at these sites releases the dsDNA fragment from the baculovirus genome, allowing its insertion in the genome of the transduced target cells. Following transduction with all-in-one HDR- or HITI-2c-pBV, HEK293T were analysed for mCherry fluorescence by flow-cytometry 10 days post-transduction and taken as a measure of successful integration (Figure 4C,D). Both HDR and HITI-2c-pBVs successfully generated bona-fide knock-in events, resulting in 0.77% and 1.71% mCherry positive cells, respectively (Figure 4D). To confirm precise integration, transduced cells were puromycin selected and genotyped to verify the presence of the predicted 5′ junction (Figure 4E). Since the success rate of HITI-2c relies on successive rounds of integration and excision to correct for wrongly oriented inserts (64), we hypothesized that higher Cas9 levels may increase its efficiency. To test this hypothesis, we sought to decouple donor and Cas9-pBV delivery using two separate vectors (Supplementary Figure S4A). In this manner, their ratios could be adjusted in co-transduction experiments to ultimately increase knock-in efficiencies. Indeed, these were increased by ∼2.3-fold when the ratio of Cas9-pBV to Donor-BV was adjusted to 2:1 (Supplementary Figure S4B,C). Although effective, co-transduction approaches eliminate the advantage of having all-in-one pBVs, which are particularly practical from a manufacturing and experimental point of view.

For this reason, we next investigated whether, by using the selective protein loading strategy already implemented in spBVs, we could generate all-in-one HITI-2c vectors capable of supporting higher knock-in rates. To this end, we generated a HITI-2c cassette for ACTB C-terminal tagging with mCherry::T2A::Puro (Figure 4F), which enables qualitative assessment of knock-in events by monitoring mCherry subcellular localization (34). As previously, HITI-2c donors and *ACTB* sgRNAs expression cassettes were combined in a single vector, with modules for selective Cas9 incorporation, to generate all-in-one HITI-2c-spBVs (Figure 4F). HeLa, A549, RPE1-hTERT, SH-SY5Y and HEK293T were transduced with increasing doses of HITI-2c spBVs amplified in the presence or absence of 100 μM ABA. At 48 h post-transduction, transduction efficiencies (eGFP expression) (Supplementary Figure S4D), knock-in efficiencies (mCherry expression) (Figure 4G) and viability (DRAQ7- events) were monitored (Supplementary Figure S4E). All the cell lines displayed dose-dependent generation of bona-fide knock-in events, with a marked increase in editing rates for HITI-2c-spBVs amplified in the presence of ABA (Figure 4G). Peak knock-in efficiencies were observed for HITI-2c spBV ABA transduced cells, albeit at different rates (HeLa ≈ 0.4%, A549 ≈ 1%, RPE1-hTERT ≈ 4%, SH-SY5Y ≈ 3.5% and HEK293T ≈ 5%). Of note, in poorly transduced cell lines (e.g. HeLa), editing events were only detected for HITI-2c-spBVs cultured in ABA, highlighting the beneficial effect of the selective protein cargo loading strategy. Once again, HITI-2c-spBVs were well tolerated, with the exception of A549 (for which a dose-dependent decrease in viability was observed), HeLa and RPE1-hTERT (for which the highest dose sensibly decreased viability) (Supplementary Figure S4E).

Live-cell confocal imaging of all transduced cell lines revealed cytoplasmic mCherry fluorescence excluded from the nuclear compartments, in line with the expected ACT::mCherry subcellular localisation pattern (Figure 4H). Next, all transduced cells (with the exception of RPE1-hTERT, which are already puromycin resistant) were puromycin selected for 4 days and expanded in the absence of antibiotic selective pressure to confirm the persistence of the integrated transgene. Transiently selected cell lines displayed homogenous mCherry expression, with the expected subcellular localisation pattern (Supplementary Figure S4F).

In all cases, PCR genotype on the selected cells (or unselected RPE1-hTERT), revealed the predicted 5′ and 3′ junctions at the integration site, confirming faithful integration of the HITI-2c donor at the *ACTB* locus (Figure 4I).

Next, we proceeded to test HITI-2c-spBV in additional cell lines, including Jurkat (T-cell leukemia), H4 (neuroglioma), primary Huvec (human umbilical vein endothelial cells) and peripheral blood mononuclear cells (PBMCs). With the exception of PBMCs, for which no transduction or editing events were recorded (data not shown), HITI-2c spBVs successfully induced the generation of knock-in events, albeit at extremely low rates. For instance, Jurkat displayed no DNA delivery, but detectable knock-in events with a peak efficiency of ≈ 0.03% at low viral doses (Supplementary Figure S4G and H, respectively). In contrast H4, while having an average DNA delivery efficiency of 35%, had similarly low rates of knock-in (Supplementary Figure S4I and J, respectively). Despite the low knock-in rates, both Jurkat and H4 ACTB::mCherry+ cells could be expanded after transient puromycin selection, confirming both actin-like mCherry subcellular localisation and presence of the expected 5′ and 3′ junctions (Figure 4J-M). Finally, eGFP expression and editing events were also detected in primary Huvec, albeit at extremely low rates (Supplementary Figure S4K–M). Furthermore, eGFP expression decreased when higher viral doses were used (Supplementary Figure S4L), potentially underlying VSV-G induced toxic effects. Similarly to Jurkat cells, the knock-in events (0.05% peak efficiency), were only observed at lower viral doses (Supplementary Figure S4M). In all cases however, higher knock-in rates were once again observed when viruses were amplified in the presence of ABA (Supplementary Figure S4H, J and M).

Taken together, these results demonstrate that spBVs can be used to simultaneously deliver Cas9 protein alongside genetically encoded sgRNAs and HITI-2c DNA donors. Encouragingly, these viruses can elicit targeted gene insertion in a range of human cells with reasonable efficiencies. While this constitutes an advancement in targeted DNA integration using protein and DNA co-delivery, these experiments also highlight inherent cell-type knock-in efficiencies upon HITI-2c spBV transduction.

## Discussion

Improving delivery and eliminating off-target activity are two strongly interdependent and critical objectives for bench-to-bedside translation of CRISPR therapies. Unfortunately, many current delivery approaches are at odds with efforts to mitigate off-target effects. Viral vectors, coupled with DSB generation, crucially pose the risk of insertional mutagenesis (34,65,66), with their inherently prolonged gene expression further increasing off-target editing (10). Protein delivery seeks to minimize off-target effects by limiting the circulation time of active Cas9. These findings led to a fast-growing number of new protein delivery vectors tailored for genome editing. Amongst others, promising approaches include VLPs (26,32,67), lentivirus-based delivery of SpCas9 fused to HIV-1 (31), ‘nanoblades’ consisting of engineered murine leukaemia VLPs loaded with Cas9-sgRNA RNPs (28) and an engineered bacterial contractile injection system (68). Importantly however, current approaches remain restricted in terms of heterologous DNA cargo capacity, thereby narrowing their scope of intervention to gene disruption or base conversions. Baculoviruses, in contrast, have unparalleled DNA cargo capacity and the potential to overcome current limitations in protein and DNA co-delivery. Here we demonstrate efficient, programmable delivery of recombinant, functional proteins into human cells using baculovirions engineered for this purpose. Our approach can thus provide a platform that satisfies the unmet need for a viral vector compatible with simultaneous co-delivery of proteins and large, heterologous DNA payloads.

Baculoviruses have been widely used as recombinant protein expression or gene delivery vectors in insect and mammalian cells, respectively (34,37–39,41). Pioneer work from Carbonell and Miller (1987) (49) provided the first observation of BV-mediated protein transfer to mammalian cells. Nonetheless, protein delivery using baculovirus vectors has remained largely unexplored to date except a very recent report (69) while applications, detailed mechanisms of protein incorporation and delivery are not explored in these studies (49,69).

We hypothesised that, during viral budding, highly expressed cytoplasmic proteins could be stochastically entrapped in previously identified viral pockets (48), a hollow space between the nucleocapsid and its surrounding envelope.

This was encouraged by previously published BV proteomics data, which identified several viral and insect-cell host proteins not apparently associated with either BV or the nucleocapsid structures (50,51), reinforcing the idea that soluble cytoplasmic proteins could be randomly stripped and encapsulated in budding virions.

To validate and characterise protein-delivery via BV (pBV) we engineered vectors for simultaneous delivery of protein and DNA cargoes using a dual-fluorescent reporter system. In doing so, we demonstrated incorporation of mCherry protein into pBVs during packaging and quantified their delivery into target mammalian cells following transduction. While mCherry fluorescence was physically linked to purified pBVs, filtration severely compromised protein delivery and, to a much lesser extent, DNA delivery. This suggests that damaged viral envelopes lead to premature release of protein cargoes, restricting successful protein transfer.

Remarkably, pBVs induced fast protein uptake dynamics in target mammalian cells, with up to 100% protein delivery achieved within 4 h post-transduction. Genetically encoded fluorescence, on the other hand, had markedly slower expression dynamics, increasing until 24 h post-transduction.

Importantly, protein cargoes displayed correct subcellular localisation in target cells, suggesting that additional functions, besides fluorescence, were successfully retained following delivery.

Building on this finding, we observed that Cas9 (163 kDa), could also be successfully loaded into pBVs while retaining its editing activity. Indeed, pBV transduction with sgRNAs provided in trans gave significantly higher editing activity in target cells than standard plasmid transfection protocols. Further analysis by UPLC-MS/MS showed that Cas9 protein levels were amongst the highest of all proteins detected in pBVs, demonstrating the inherent protein-loading capabilities of pBVs. Similarly to previous reports (49,50), we also detected highly expressed cytosolic insect cell host proteins in pBVs, suggesting a correlation between protein expression levels in the cytoplasm and their relative abundance in the baculovirions. The entrapment and delivery of unintended insect host proteins to target cells is unlikely to impact on the safety and efficacy of the baculovirus as a delivery vector, as supported by their low cytoxicity in mammalian cells (70). Similarly, delivery of stochastically entrapped host cytoplasmic proteins is likely affecting other protein delivery systems, for instance VLPs produced in mammalian cells (27,28,31,62,67).

We demonstrate that Cas9 delivery via pBV resulted in high levels of mCherry KO when sgRNAs were provided in trans, markedly outcompeting plasmid transfection in our hands. More importantly, pBV DNA cargoes could be further engineered to host in *cis* sgRNAs expression. In contrast to plasmid co-transfection, this approach resulted in higher gene editing efficiencies (up to 95% indels), with overall faster dynamics (from 24 h post-transduction). Although Cas9 protein delivery should be maximally internalised between 4 and 8 h post-transduction, no indels were observed earlier than 24 h post-transduction. Indeed, while Cas9 transcriptional and translational latency were eliminated via protein delivery, the *cis* acting sgRNA is still subject to transcriptional latency.

For this reason, we hypothesised that pBVs could be further engineered to deliver Cas9 and sgRNAs ribonucleoprotein complexes (RNPs). The generation of RNP-pBVs would be highly desirable not only to grant even faster editing dynamics, but also to extend gene editing applications to cell types in which BV-mediated gene delivery is subject to silencing (71–73).

Although they were successfully produced, RNP-pBVs displayed overall lower editing efficiencies than Cas9-pBVs outfitted with a human sgRNA expression cassette. We also demonstrated that poor sgRNA packaging was the cause of the reduced editing activity, potentially suggesting that nuclear localisation of SfU6 transcripts is not fully compatible with cytoplasmic Cas9 loading into pBV. Notwithstanding, this is the first example of a baculovirus simultaneously delivering heterologous DNA, protein and RNA to human cells although further work will be required to fully characterise and engineer more efficient RNP-pBVs.

In addition to Cas9, efficient delivery of a much larger base editor (BE3, 193 kDa) protein was also possible via pBV equipped with in *cis* sgRNAs cassettes, resulting in high levels of base conversion in target cells. These data further confirmed that pBV-based protein delivery appears to be unrestricted by protein cargo size.

Encouraged by these results, we aimed to selectively incorporate protein cargoes into BVs, rather than relying on stochastic protein entrapment. While nucleocapsid tethering strategies in which Cas9 was fused to structural nucleocapsid proteins (P6.9 or VP39) were successful, gene editing levels in target cells were compromised. In marked contrast, selective tethering to VSV-G using chemically-inducible heterodimerization successfully improved both Cas9 protein loading and delivery efficiencies, resulting in higher gene editing rates. Selective protein-loaded BVs (spBVs), markedly increased protein delivery and editing efficiencies at much lower doses compared to standard pBVs, while also enabling multiplexed gene editing in a panel of human cell lines. Importantly, despite their higher protein delivery and on-target editing efficiencies, Cas9-spBVs did not induce detectable editing at off-target sites, in contrast to genetically encoded Cas9 delivery.

Finally, we engineered all-in-one spBVs for co-delivery of Cas9 protein and genetically encoded sgRNAs and DNA donor cassettes for homology-independent targeted integration (HITI-2c). Indeed, knock-in of large DNA fragments remains particularly challenging when coupled with RNPs or Cas9 protein delivery (28). However, HITI-2c spBVs were able to induce knock-in events in all the transduced cell lines, with the exception of PBMCs. Successful knock-in rates (up to 5%) remained highly cell-type dependent and did not entirely correlate with DNA transduction efficiencies. For instance, while no CMV eGFP expression was detected in Jurkat, rare knock-in events were observed, suggesting that the Cas9 protein and sgRNAs expression were functional, at least to some extent. In contrast, low editing rates were observed in H4 and primary Huvec, despite successful transduction. This indicates potential issues with Cas9 internalisation and activity and/or sgRNA expression following transduction. This is especially evident in primary cell types and further optimisation will be required to increase efficiencies in these cells. Differences in transduction efficiency, protein trafficking and/or other cell-specific innate immunity mechanisms may also underlie these observations, suggesting that further investigation or vector engineering may be required in the future.

Nonetheless, these data illustrate the ability of spBVs to deliver complex CRISPR editing toolkits alongside Cas9 protein in a range of human cell lines.

Although editing efficiencies could potentially be improved by increasing spBV dosage, VSV-G dependent cytotoxicity (63), prevents application of extremely large multiplicities of transduction. In line with this, we observed a detrimental effect of increased viral dosage on viability (e.g. RPE1-hTERT, A549) or DNA delivery efficiency (Huvec) in a subset of sensitive cell lines. In the future, decoupling selective Cas9 loading from pseudotyping modules, could effectively increase protein delivery and editing efficiencies in a wider range of target cells, while having reduced cytotoxic effects.

Taken together, we highlight the ability of protein-loaded baculovirions to achieve precision genome editing using various CRISPR-based techniques. Our work now opens a range of interesting possibilities for future applications. For instance, addition of a dominant negative mismatch repair protein (MLH1dn) (74) could be used to effectively boost prime editing efficiencies. Similarly, pBVs could be equipped for the delivery of recombinases, integrases and transposases, or combinations thereof, to realize a range of genetic modifications and interventions simultaneously (5,6,75).

This approach unlocks a hitherto largely unexplored capability of baculoviruses and greatly expands their versatility as a viral vector. By combining the safety of protein-based delivery with the advantages of the baculovirus, we believe that pBVs and spBVs could significantly contribute to improve safety, efficiency and scope of current gene editing interventions.

## Supplementary Material

gkae142_Supplemental_Files

## Data Availability

All plasmid sequences are provided in Supplementary Table ST1 and Supplementary Information. Sequences of all the sgRNA and PCR oligonucleotides are provided in Supplementary Table ST2 and ST3, respectively. Raw proteomics data are provided in Supplementary Table ST4. All other reagents are available from the authors upon reasonable request. Raw flow-cytometry and Sanger sequencing data have been deposited under the following DOI: https://doi.org/10.6084/m9.figshare.24950637.

## Notes

Present address: Julien Capin, Centre de Biologie Structurale, INSERM U1054, CNRS UMR5048, University of Montpellier, 34090 Montpellier, France.
