# Supplementary material for: An engineered baculoviral protein and DNA co-delivery system for CRISPR-based mammalian genome editing
Source: Nucleic Acids Res. 2024 Feb 27;52(6):3450–68. doi: 10.1093/nar/gkae142 (PMC11014373; doi:10.1093/nar/gkae142)
Supplement: gkae142_Supplemental_Files [file gkae142_supplemental_files.zip › Supplementary_data_Capin_et_al_with_figures.pdf]

An engineered baculoviral protein and DNA co-delivery system for CRISPR-based mammalian genome editing

Julien Capin[1†], Alexandra Harrison[1†], Renata A. Raele[1], Sathish K. N. Yadav[1], Dominique Baiwir[4], Gabriel Mazzucchelli[5], Loic Quinton[5], Timothy J. Satchwell[1], Ashley M. Toye[1], Christiane Schaffitzel[1], Imre Berger[1,2,3]*†† and Francesco Aulicino[1]*††

[1] School of Biochemistry, University of Bristol, 1 Tankard's Close, Bristol BS8 1TD, UK.

[2] School of Chemistry, University of Bristol, Cantock's Close, Bristol BS8 1TS, UK.

[3] Max Planck Bristol Centre for Minimal Biology, Cantock's Close, Bristol BS8 1TS, UK

[4] GIGA Proteomics Facility, University of Liege, B-4000 Liege, Belgium

[5] Mass Spectrometry Laboratory, MolSys Research Unit, University of Liège, 4000, Liège, Belgium

* To whom correspondence may be addressed: imre.berger@bristol.ac.uk +44 79 0720 8697

Correspondence may also be addressed to francesco.aulicino@bristol.ac.uk +44 117 394 1251

† Joint first-authors

†† Joint last-authors

Present address: Julien Capin, Centre de Biologie Structurale, INSERM U1054, CNRS UMR5048, University of Montpellier, 34090 Montpellier, France

List of contents

Supplementary Figure S1. Characterisation of protein-loaded BV (pBV) 3

Supplementary Figure S. Characterisation of Cas9-pBV 5

Supplementary Figure S3 – Selective Cas9 protein loading strategies in pBV........ 7

Supplementary Figure S4 - Knock-in using dual, all-in-one pBV or spBV approaches across different cell types........ 9

Supplementary Table ST1 – Plasmid sequences and assembly information, provided as an Excel spreadsheet 11

Supplementary Table ST2 – List of sgRNAs used in this study........ 11

Supplementary Table ST3 – List of oligonucleotides used in this study 11

Supplementary Table ST4 - UPLC-ESI-MS/MS results. Provided as an Excel spreadsheet 12

Supplementary plasmids maps and plain text sequences........ 12

Supplementary Figure S1. Characterisation of protein-loaded BV (pBV)

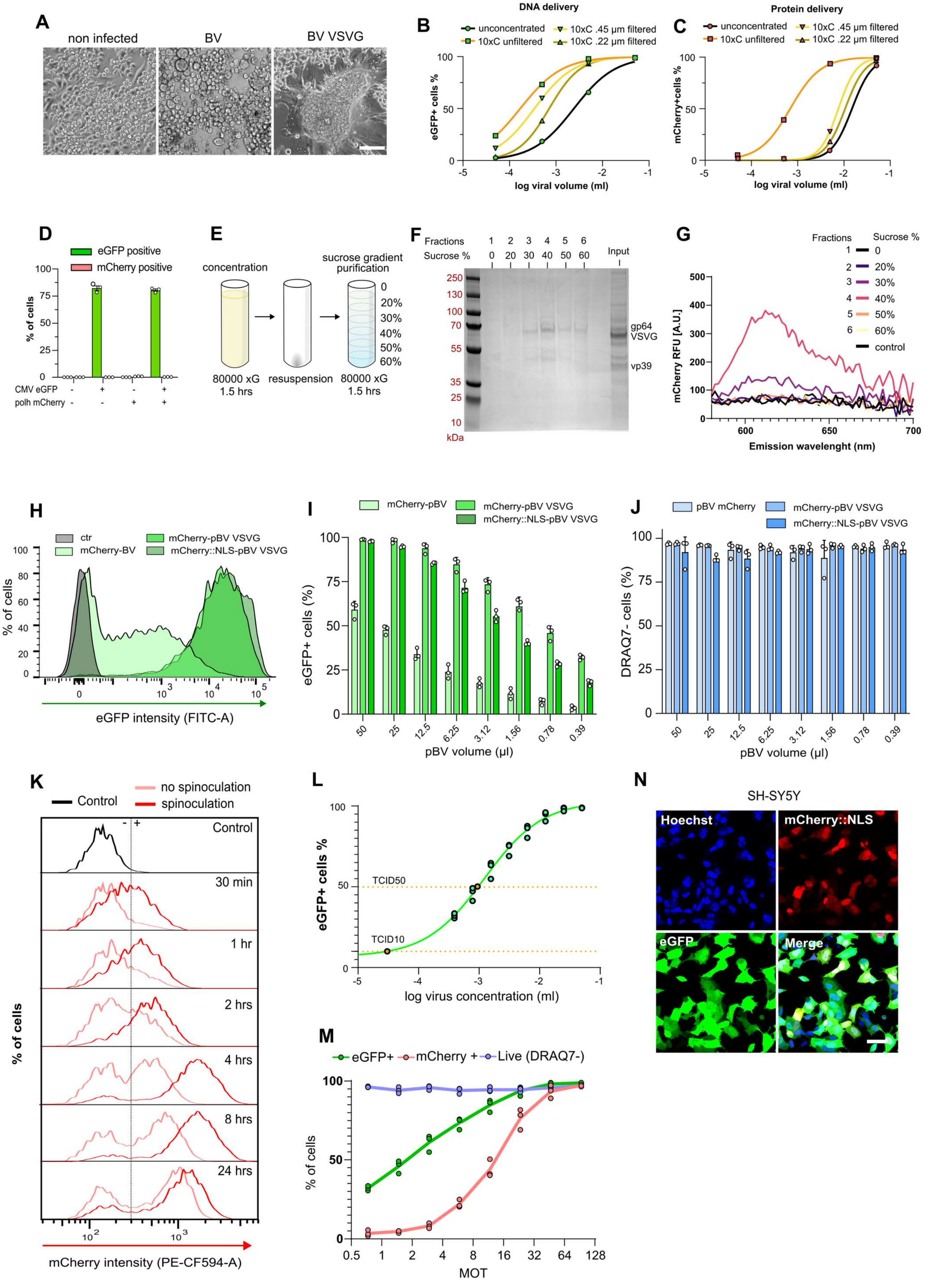

(**A**) Live widefield microscopy of Sf21 cells morphology under normal culturing conditions (non infected) or during viral amplification in presence or absence of VSVG pseudotyping modules (BV and BV VSVG, respectively). Scalebar is 50 µm. (**B**) DNA (eGFP+ %) and (**C**) protein (mCherry+ %) delivery efficiencies in HEK239T transduced with serially diluted mCherry::NLS-pBV VSVG. Crude viral supernatant (unconcentrated) or 10-fold concentrated and filtered virus were used. Flow-cytometry data of one representative experiment. (**D**) eGFP and mCherry detection in HEK293T analysed at 48 hours after transfection with the indicated plasmids. Data are mean ± s.d. of n = 3 independent biological replicates analysed by flow-cytometry. (**E-G**) Validation of protein cargo presence in purified mCherry-pBV VSVG. (**E**) Schematic representation of concentration and purification by sucrose gradient ultracentrifugation; (**F**) Representative Coomassie staining of SDS-PAGE gel loaded with 20 µl of each fraction (gp64, VSVG and vp39 are identified based on molecular weight). Input is concentrated, unprocessed virus. (**G**) Representative plate-reader mCherry spectral scanning in 50 µl of each sucrose fraction. Control is PBS. (**H-J**) eGFP expression and viability of HEK293T 24 hours after transduction with the indicated pBV (relative to Fig.1 D). (**H**) Representative eGFP flow-cytometry histograms of 10x concentrated pBV, (**I**) percentages of eGFP+ cells and (**J**) DRAQ7- cells (viable cells) upon serial-dilutions of concentrated viral stocks. Data are mean + s.d. of n = 3 independent biological replicates analysed by flow-cytometry. Multiplicity of infections of undiluted pBV are: mCherry-pBV≈1, mCherry-pBV VSVG≈ 50 and mCherry::NLS-pBV VSVG≈ 20. (**K**) Representative mCherry flow-cytometry histograms, relative to Fig.1E,F. (**L**) Exemplificative fluorescence based estimation of viral transducing units (TU) following serial dilution and transduction of HEK293T. (**M**) Plot of eGFP+, mCherry+ and DRAQ7- (viable) HEK293T cells at 24 hours post-transduction with mCherry-pBV VSVG at the indicated multiplicity of transductions (MOTs). Flow-cytometry data of n=3 independent biological replicates. (**N**) Live confocal microscopy of SH-SY5Y cells at 48 hours post-transduction with pBV NLS-mCherry at MOT = 10. Hoechst dye counterstains nuclei. Scalebar is 50 µm.

Supplementary Figure S. Characterisation of Cas9-pBV

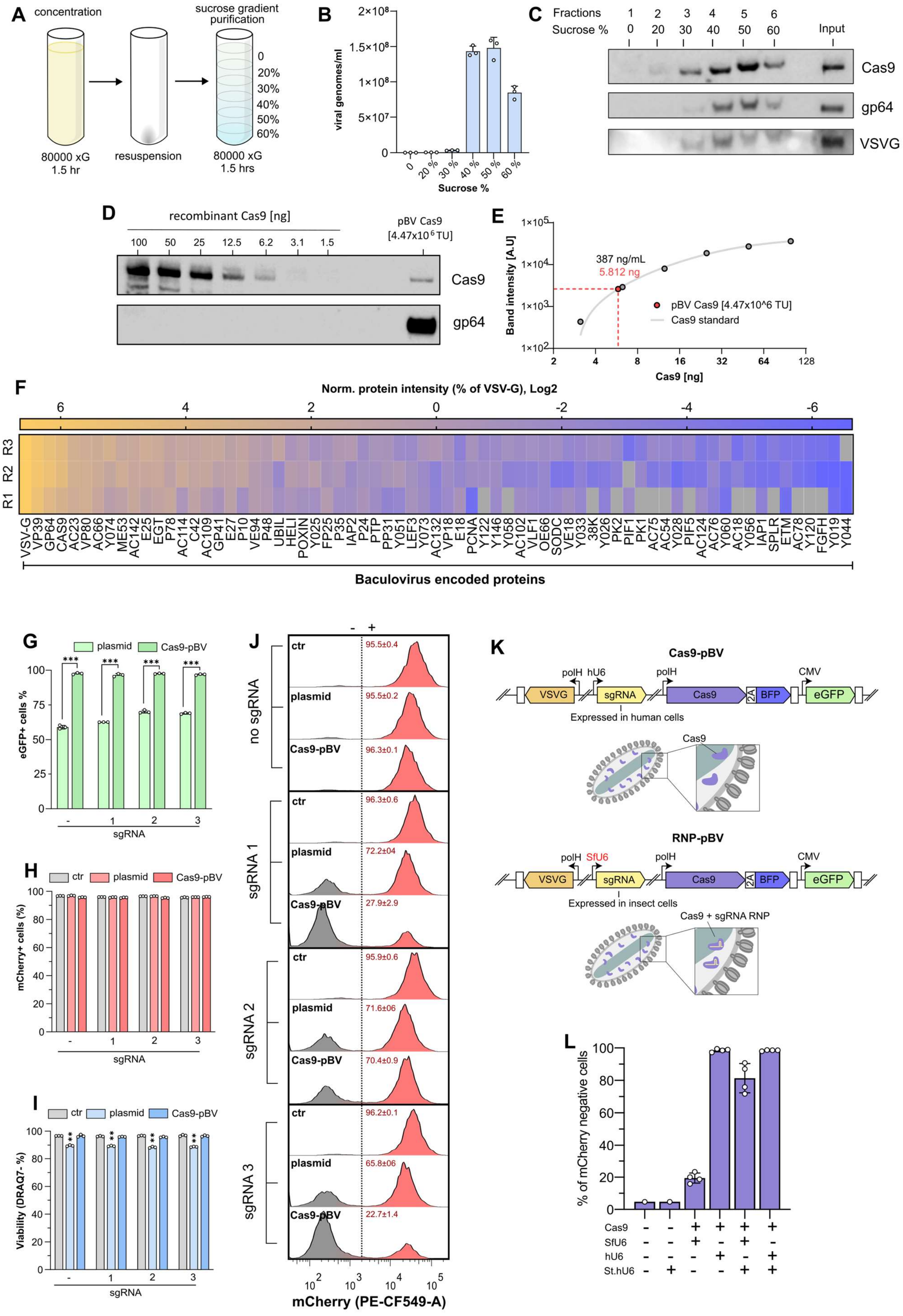

(**A-C**) Cas9-pBV purification and characterisation. (**A**) Schematic representation of concentration and purification by sucrose gradient ultracentrifugation. (**B**) Representative estimation of viral genome copies per ml (vg/ml) via qPCR in the indicated sucrose fractions. data are mean + s.d. of n=3 technical replicates. (**C**) Western blot of Cas9, gp64 and VSVG across the indicated fractions. (**D-E**) Estimation of Cas9 mass in concentrated Cas9-pBV using western blot against a known purified Cas9 standard (**D**) followed by band intensity quantification and and (**E**) standard curve calibration. (**F**) Heatmap of normalised viral proteins intensities detected by UPLC-MS/MS in concentrated Cas9-pBV (relative to Fig 2B). Protein intensities across samples have been normalised to spiked in proteins and are ranked from left to right according to their abundance relative to the most abundant protein: VSVG. R1, R2 and R3 are three biological replicates. Grey box indicate that the protein was not detected in the corresponding sample. (**G-I**) eGFP+ (**G**), mCherry+(**H**) and DRAQ7- (**I**) cells percentages 48 hours after transfection or 24 hours after transduction of HEK293T stably expressing mCherry and sgRNAs with plasmid (500 ng) or Cas9-pBV (MOT 50) (relative to Fig.2 D). Histograms of flow-cytometry data. Mean ± s.d. of n = 3 independent biological replicates. ** $P < 0.01$, Student's t-test. (**J**) Representative flow-cytometry histograms of HEK293T stably expressing mCherry and sgRNAs 7 days post-transfection or transduction with Cas9 encoding plasmid (500 ng) or Cas9-pBV (MOT 50). (Relative to Fig.2 D). (**K-L**) mCherry KO with RNP-pBV in HEK293T stably expressing mCherry. (**K**) Constructs for Cas9-pBV or RNP-pBV production. SfU6 = Spodoptera frugiperda U6 promoter; hU6 = human U6 promoter. (**L**) mCherry knock-out efficiencies at 10 days post-transduction with pBV carrying the indicated elements in HEK293T stably expressing mCherry. A stable cell line expressing mCherry and sgRNA (St.hU6) was additionally used as control. Histogram of flow-cytometry data. mean ± s.d. of n = 4 independent biological replicates.

Supplementary Figure S3 – Selective Cas9 protein loading strategies in pBV

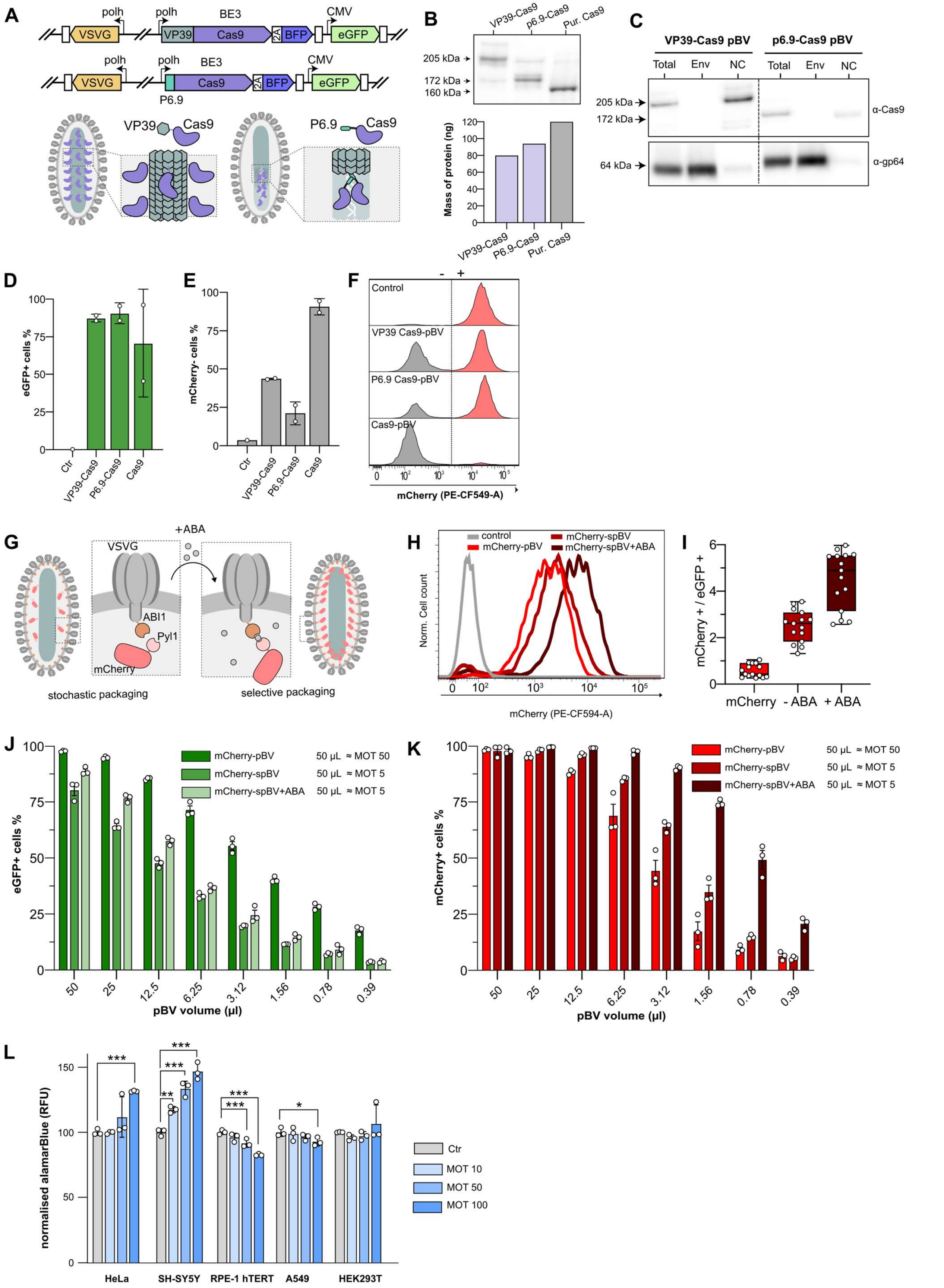

(**A**-**C**) Testing of Cas9 protein delivery via tethering to structural nucleocapsid components. (**A**) Schematic representation of the constructs (top) and predicted Cas9 intra-viral localization (bottom) following generation of VP39 and P6.9 Cas9 fusion proteins. (**B**) Representative western blot against Cas9 on concentrated viral stocks produced with the indicated constructs (top) and band intensity quantification (bottom). A purified Cas9 with known mass is used as control. (**C**) Western blot of GP64 (major BV glycoprotein) and Cas9 on NP-40 fractionated viruses. Viruses were concentrated and purified by discontinous sucrose gradient ultracentrifugation. Nucleocapsid and envelopes were separated via NP-40 incubation, followed by a second discontinous sucrose gradient ultracentrifugation. Env = envelope fraction, NC = nucleocapsid fraction. (**D**-**F**) mCherry knock-out efficiencies in HEK293T stably expressing mCherry and sgRNA 3 using nucleocapsid tethered Cas9 pBV described in (A) . (**D**) DNA delivery efficiency and mCherry KO rates at 24 hours and 10 days post-transduction, respectively. MOT = 50 for all the indicated pBV. Histograms of flow-cytometry data. Mean ± s.d. of n=2 independent biological replicates. (**F**) Representative flow-cytometry histograms of mCherry loss at 10 days post-transduction, relative to (E). (**G**-**K**) Validation of abscisic acid (ABA) inducible selective mCherry protein packaging strategy. (**G**) Pyl1 and ABI1 domains are fused to the N-terminal and C-terminal domain of mCherry and VSVG, respectively. During viral packaging, addition of ABA promotes selective packaging of mCherry into budded virions. (**H**-**K**) mCherry protein incorporation in HEK293T transduced with standard (pBV) or selective (spBV) protein delivery BV amplified in presence or absence of 100 μM ABA. (**H**) Representative flow-cytometry histograms at 24 hours post-transduction with MOT 50 (pBV) or 5 (spBV). (**I**) Box and whiskers plot of the ratios of eGFP/mCherry positive cells obtained within the linear dilution range (1:8 – 1:128) obtained with the indicated vectors. n = 15 independent transductions, relative to (J and K). (**J**) DNA (eGFP) and (**K**) protein (mCherry) delivery efficiencies in HEK293T at 24 hours post-transduction with serially diluted pBV or spBV. Undiluted virus MOT are 50 (mCherry-pBV) and 5 (mCherry-spBV with or without ABA). Histograms of flow cytometry data, mean ± s.d. of n=3 independent biological replicates. (**L**) Alamar blue staining quantification in a panel of human cell lines at 48 hours post-transduction with a dual Cas9-spBV at the indicated MOTs. Relative to Figure 3 (I-K). Data are mean ± s.d. of plate reader fluorescence measurment. n=3 independent biological replicates. *** P <0.001, ** P< 0.01, * P <0.05, Student's *t*-test.

Supplementary Figure S4 - Knock-in using dual, all-in-one pBV or spBV approaches across different cell types

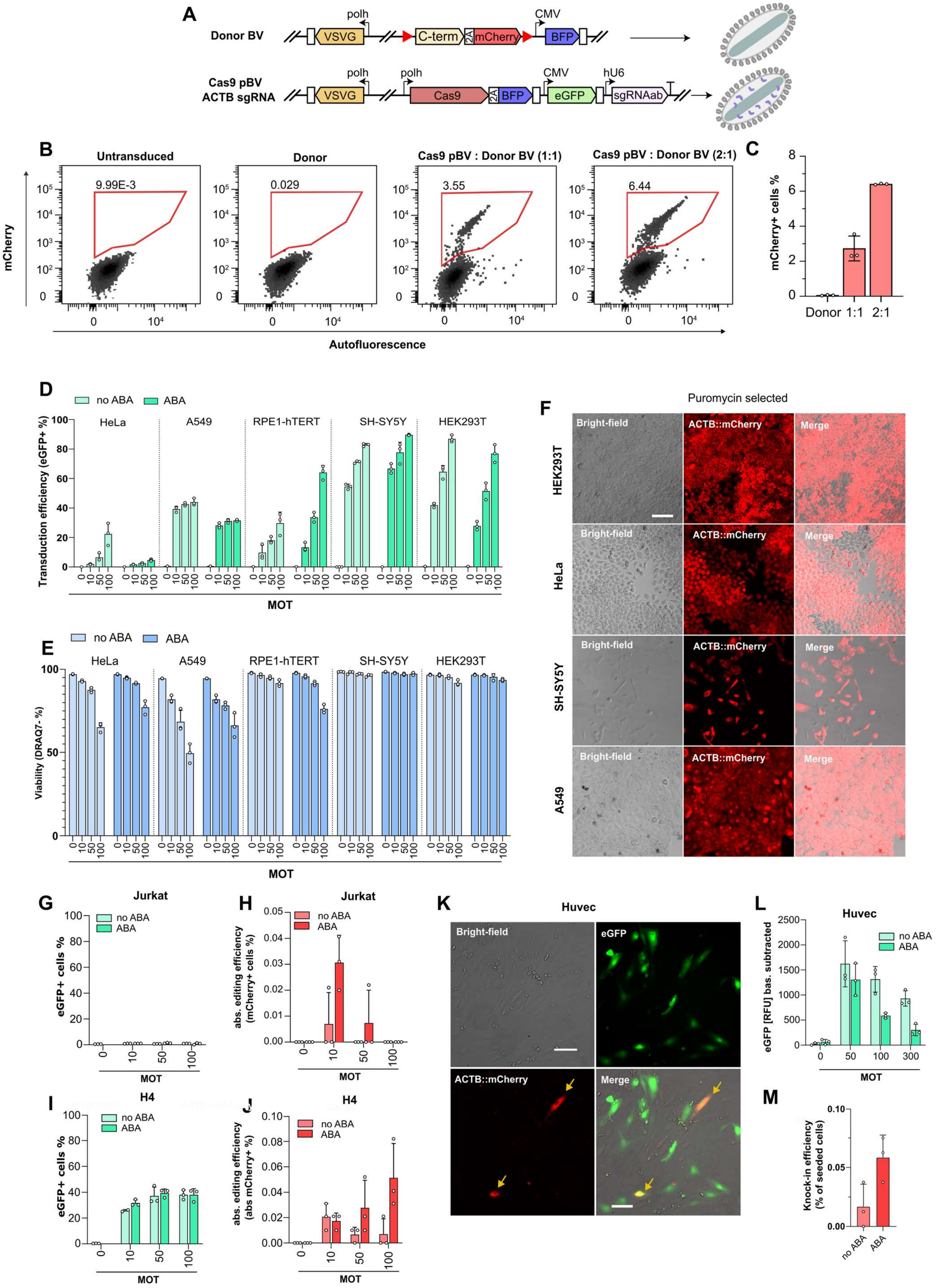

(**A-C**) Separate Cas9-pBV and donor BV transduction strategy to increase knock-in efficiency. (A) Schematic representation of the construct used to produce individual HITI-2c donor BV and Cas9-pBV. (**B-C**) Knock-in efficiencies (mCherry + cells) in HEK293T at 5 days post co-transduction with the indicated ratios of Cas9-pBV and HITI-2c donor BV (MOT/MOT 1=100). (**B**) Representative flow-cytometry dot plots and (**C**) histogram of flow-cytometry data. mean ± s.d. of n=3 independent biological replicates. (**D-E**) Transduction efficiency (eGFP+) (**D**) and Viability (DRAQ7-) (**E**) percentages of HeLa, A549, RPE-1 hTERT, SH-SY5Y and HEK293T at 48 hours post-transduction with HITI-2c spBV amplified in presence or absence of 100 µM ABA at the indicated MOTs. Data are mean + s.d. of flow-cytometry data, n=3 independent biological replicates. Relative to Fig. 4 G. (**F**) Representative live confocal imaging of HEK293T, HeLa, SH-SY5Y and A549 transduced with HITI-2c spBV ABA at MOT 100 after puromycin selection for 4 days and amplification in absence of selective pressure for 15 days. Scalebar is 100 µm. Relative to Fig. 4G-I. (**G-J**) Transduction (eGFP+) and knock-in (mCherry+) efficiencies in Jurkat (**G-H**) and H4 cell lines (**I-J**) at 48 hours post-transduction with the indicated spBV at different MOTs. Relative to Fig. 4J-M. Data are mean + s.d. of flow-cytometry data, n=3 independent biological replicates. (**K-M**) Knock-in in primary Huvec via HITI-2c spBV. (**K**) Live widefield fluorescence microscopy at 4 days post-transduction with HITI-2c spBV ABA MOT 50. Scalebar is 100 µm. Yellow arrows indicate edited cells. (**L**) eGFP expression levels 24 hours post-transduction with the indicated spBV at different MOTs. Mean ± s.d. of plate reader eGFP fluorescence measurements. n = 3 independent biological replicates. (**M**) Knock-in efficiencies at 4 days post-transduction following transduction with HITI-2c spBV at MOT 50. Mean + s.d. of microscopy counts of n=3 independent transductions.

Supplementary Table ST1 – Plasmid sequences and assembly information, provided as an Excel spreadsheet

Supplementary Table ST2 – List of sgRNAs used in this study

sgRNAs

Name	Target	Spacer sequence (5'-3')	Figures	Source
sgRNA#1	mCherry	CTCCGAGCGGATGTACCCCG	Fig2	This study
sgRNA#2	mCherry	GAACTTCGAGGACGGCGGCG	Fig2	This study
sgRNA#3	mCherry	GGAGCCGTACATGAACTGAG	Fig2	This study
hACTB sgRNA	B-Actin	GACAGCTCCCCACACACCAC	Fig4	Aulicino et al. 2022
BE3 sgRNA	mCherry	TGTCCCAGGCGAAGGGCAGG	Fig2	Kuscu et al. 2017
HEKs1 sgRNA	HEKs1	GGGAAAGACCCAGCATCCGT	Fig3	Indikova et al. 2020
HEKs3 sgRNA	HEKs3	GGCCCAGACTGAGCACGTGA	Fig3	Indikova et al. 2020
EMX1 sgRNA	EMX1	GAGTCCGAGCAGAAGAAGAA	Fig3	Gee et al. 2020
VEGFA sgRNA	VEGFA	GGTGAGTGAGTGTGTGCGTG	Fig3	Gee et al. 2020

Supplementary Table ST3 – List of oligonucleotides used in this study

Name	Locus	Sequence (5'-3')	Use	Figures
sgRNA3-mcherry-For	mCherry	TCTGCTAACATGCGGTGACG	gPCR, ICE	Fig2
sgRNA3-mcherry-Rev	mCherry	TCCATGCCGCCGGTGGAGTG	gPCR	Fig2
hACTB-WT-For (For1)	ACTB	ATGAAGATCAAGGTGGGTGTCTTT	gPCR	Fig4
hACTB-WT-Rev (Rev1)	ACTB	TCTCAAGTCAGTGTACAGGTAAGC	gPCR	Fig4
hACTB-KI-mcherry-Rev (5′ junction) (Rev2)	ACTB-mCherry KI (5′)	CAAGTAGTCGGGGATGTCGG	gPCR	Fig4
hACTB-KI-mCherry-For (3′ junction) (For2)	ACTB-mCherry KI (3′)	CAACCTCCCCTTCTACGAGC	gPCR	Fig 4
hACTB-KI-mCherry-Rev (3′ junction) (Rev3)	ACTB-mCherry KI (3′)	GAAGCATTTGCGGTGGACGA	gPCR	Fig 4
BE3-For	mCherry	TCGTCCACCGCAAATGCTTCGTGAGCAAGGGCGAGGAGGA	gPCR	Fig2
BE3-Rev	mCherry	TTGTACAGCTCGTCCATGCC	gPCR,EditR	Fig2
HEKs1-For	HEKs1	GCTTTCCTGCCCCAGAGTC	gPCR	Fig3G
HEKs1-Rev	HEKs1	CCTGACACTTCTTAGAATGGCCT	gPCR, ICE	Fig3G
HEKs3-For	HEKs3	ACTTTAGACCTTAGAGGCCCCAGG	gPCR	Fiig3G
HEKs3-For(2)	HEKs3	GGGTCACAGTGGCAAATGAG	gPCR	Fig3K
HEKs3-Rev	HEKs3	TGTTGAGCTCGACCCTGAAG	gPCR, ICE	Fig3G,K
EMX1-For	EMX1	CTGCCATCCCCTTCTGTGAATGT	gPCR, ICE	Fig3
EMX1-Rev	EMX1	GGAATCTACCACCCCAGGCTCT	gPCR	Fig3
EMX1off-For	EMX1 off-target	GGTTCTGTAAACGCCGTAGC	gPCR, ICE	Fig3
EMX1off-Rev	EMX1 off-target	GGAACTACAAGAATGCCTGAGC	gPCR	Fig3
VEGFA-For	VEGFA	GCATACGTGGGCTCCAACAGGT	gPCR, ICE	Fig3
VEGFA-Rev	VEGFA	CCGCAATGAAGGGGAAGCTCGA	gPCR	Fig3
VEGFAoff-For	VEGFA off-target	AATGGTAGTTGCCTGGGGATG	gPCR, ICE	Fig3
VEGFAoff-Rev	VEGFA off-target	TCTGCAGTTTTGGGTGTCTGT	gPCR	Fig3

Supplementary Table ST4 - UPLC-ESI-MS/MS results. Provided as an Excel spreadsheet

Supplementary plasmids maps and plain text sequences

Plasmids maps and plain text sequences are additionally provided as a separate .pdf document.
